# Crossover operators for molecular graphs with an application to virtual drug screening

**DOI:** 10.1186/s13321-025-00958-w

**Published:** 2025-06-17

**Authors:** Nico Domschke, Bruno J. Schmidt, Thomas Gatter, Richard Golnik, Paul Eisenhuth, Fabian Liessmann, Jens Meiler, Peter F. Stadler

**Affiliations:** 1 Bioinformatics Group, Department of Computer Science, Leipzig University, Härtelstraße 16–18 , 04107 Leipzig, Germany; 2 Interdisciplinary Center for Bioinformatics, Leipzig University, Härtelstraße 16–18, 04107 Leipzig, Germany; 3 Center for Scalable Data Analytics and Artificial Intelligence ScaDS.AI and School of Embedded Composite Artificial Intelligence SECAI, Dresden/Leipzig, Germany; 4 Institute for Drug Discovery, Leipzig University, Liebigstraße 21, 04103 Leipzig, Germany; 5 Department of Chemistry, Department of Pharmacology, Center for Structural Biology, Institute of Chemical Biology, Center for Applied Artificial Intelligence in Protein Dynamics, Vanderbilt University, 465 21st Ave S, Nashville, 37232 USA; 6 Max Planck Institute for Mathematics in the Sciences, Inselstraße 22, 04103 Leipzig, Germany; 7 Santa Fe Institute, 1399 Hyde Park Rd., Santa Fe, NM 87501 USA; 8 Facultad de Ciencias, Universidad Nacional de Colombia, Ciudad Universitaria, Sede Bogotá, Bogotá, D.C., COL-111321 Colombia; 9 Institute for Theoretical Chemistry, University of Vienna, Währingerstrasse 17, Vienna, 1090 Austria

**Keywords:** Crossover, Graph theory, Genetic algorithm, Virtual screening, 92E10, 05C30, 05C62, 05C85, 05C90

## Abstract

Genetic algorithms are a powerful method to solve optimization problems with complex cost functions over vast search spaces that rely in particular on recombining parts of previous solutions. Crossover operators play a crucial role in this context. Here, we describe a large class of these operators designed for searching over spaces of graphs. These operators are based on introducing small cuts into graphs and rejoining the resulting induced subgraphs of two parents. This form of cut-and-join crossover can be restricted in a consistent way to preserve local properties such as vertex-degrees (valency), or bond-orders, as well as global properties such as graph-theoretic planarity. In contrast to crossover on strings, cut-and-join crossover on graphs is powerful enough to ergodically explore chemical space even in the absence of mutation operators. Extensive benchmarking shows that the offspring of molecular graphs are again plausible molecules with high probability, while at the same time crossover drastically increases the diversity compared to initial molecule libraries. Moreover, desirable properties such as favorable indices of synthesizability are preserved with sufficient frequency that candidate offsprings can be filtered efficiently for such properties. As an application we utilized the cut-and-join crossover in REvoLd, a GA-based system for computer-aided drug design. In optimization runs searching for ligands binding to four different target proteins we consistently found candidate molecules with binding constants exceeding the best known binders as well as candidates found in make-on-demand libraries.

**Scientific contribution**

We define cut-and-join crossover operators on a variety of graph classes including molecular graphs. This constitutes a mathematically simple and well-characterized approach to recombination of molecules that performed very well in real-life CADD tasks.

**Supplementary Information:**

The online version contains supplementary material available at 10.1186/s13321-025-00958-w.

## Introduction

Graphs are by the far the most common mathematical formalism employed to model objects for which discrete components and their relation are an important aspect. Molecules, characterized by chemical bonds connecting atoms, were one of the earliest areas of applications already in the 19th century [1]. Nowadays, graphs are nearly ubiquitous in most areas of science and engineering, laying at the heart of the field of Network Theory [2].

Genetic algorithms (GA) are a widely used class of population-based heuristic optimization algorithms inspired by evolutionary adaptation in nature. At the core of a GA is a population of individuals that represent potential solutions to the problem at hand. In the most general setting, the population is used to produce a set of offspring by means of “genetic operators” that typically take one or two parents as input. The population of the next generation is then selected from the current population and their offspring using a selection rule that favors fitter solutions. The effectiveness of these algorithms depends on the representation of solutions, the definition of the genetic operators that act on these representations, and the selection scheme [3]. Following the paradigm of biological evolution, the overwhelming majority of GA implementations uses fixed length strings to represent solutions and employs mutation (i.e., relabeling) as well as crossover to produce variants to select from in each generation. Occasionally, other data structures are used, such as permutations as encodings of tours in the traveling salesman problem, that require specialized crossover operators [4].

Here, we aim to apply GAs to tasks that require the exploration of complex chemical spaces. Virtual Drug Screening [5] can be understood as an optimization problem over a *chemical space*, i.e., a set of molecules that may be subject to some restriction such as compliance with *Lipinski’s Rule of Five* [6]. The objective function $f:X\rightarrow {\mathbb {R}}$ estimates e.g. a binding affinity of a candidate $x\in X$ to a given drug target. In this setting, very little is known about the properties of the fitness function *f*, which is either computed from explicit molecular simulations or on the basis of a machine learning model. GAs thus are a very natural choice for the computational methodology to find candidates $x\in X$ that at least approximately optimize *f*(*x*). Evolutionary algorithms, including GAs, indeed have been used for decades in computer-aided drug discovery (CADD) [7], see [8] for a survey.

Make-on-demand libraries [9, 10] are populated *in silico* by means of fragment recombination that corresponds to easily accessible chemical reactions. Since the size of such chemical search spaces makes its enumeration impractical and in most cases impossible, GAs are a very natural approach for such problems. A simple example of such an approach is REvold [11], which is designed to optimize over the ENAMINE REAL Space [12]. In particular in the field for Virtual Drug Screening, it is of interest not only to optimize *f* but also to access molecules that are sufficiently different from well-known molecular shapes, thus are likely free from patents. This reinforces recombination of molecular fragments as an appealing search strategy.

In order to run GAs on more complex chemical spaces, the challenge is to implement efficient genetic operators that act directly on molecule graphs. As in most GA applications, we consider a mutation operator $M: X \rightarrow X$ that transforms a single candidate into a closely related variant, thus supporting local fine-tuning. Complementarily, crossover operators $C: X\times X\rightarrow X$ are meant to “interpolate” between a pair of parents, allowing the recombination of favorable features of both parents in a large “jump” in chemical space. The use of GAs in chemical space exploration, therefore, requires mutation and recombination operators that ensure that offspring are again plausible molecules. The use of exclusively mutation or recombination may also be suitable depending on definitions. A wide variety of representations have been used to represent molecules in evolutionary algorithms, including binary strings, fragments, SMILES, and of course molecular graphs.

Molecular graphs, albeit not formally defined in strict mathematical sense, are a very restricted class compared to labeled graphs in general. This is, of course, a direct consequence of the valence rules of chemistry and the fact that molecules are embedded in 3D space such that all the bond lengths are determined by bond order and the incident atoms. In particular, the structural formulae of real molecules are graph-theoretic planar graphs, i.e. they can be embedded in a plane even though the actual compound has a 3D structure, except for a small set of exceptional cases that have been synthesized mostly as curiosities [13–15].

Two classes of graph-based crossover are described in [16]: multipoint crossover starts from a cut that is chosen to minimize topological disruption and a reconnection procedure that ensures connectedness of the children [17]. Subgraph crossover, in contrast, identifies a subgraph in each parent and then proceeds by reconnecting them. In MEGA [18], crossover is guided by retro-synthetic information obtained from RECAP [19] or affects single bonds or rings [17], see also [20]. The graph-based GA described in [21] uses reaction SMILES to implement its crossover operator. EvoMD [22], finally, is defined on the binary adjacency matrices of the parent graphs. Even though graph-based GAs are not at all uncommon, very little is known about the mathematical properties of these crossover operators. Other methods have been proposed that are based solely on string manipulation. FASMIFRA [23] operates directly on SMILES, but disallows the crossover of rings. MolFinder [24] follows a similar strategy but allows crossover on rings with heuristic joining of fragments. In STONED [25] crossover and mutation based on string edit distance was proposed.

The main objective of this contribution is to define and characterize a large class of crossover operators for graphs. The key idea is to use cuts in graphs as means of (bi-)partitioning the parents and to recombine the resulting parts by means of a join operation. This very general scheme of cut-and-join crossover subsumes most of the procedures in graph-based GAs applied to chemical space exploration. Instead of implementing specific restrictions motivated by chemical intuition, we start from a very general construction that is not related to chemistry. We proceed to demonstrate that this leads to a class of operators that can be tuned in a very natural fashion to preserve both local properties, such as vertex degrees, as well as certain global properties, such as graph-theoretic planarity, that are of direct relevance for applications in chemistry. In other words, the proposed method is agnostic to chemical properties, but conserves them naturally without artificial restrictions. Our method was conceived as a well defined mathematical framework. Contrasting to previous publications, this framework yields simple to proof guarantees on which compounds can be generated, including synthesis paths. It has the potential to proof convergence for specific applications of genetic algorithms on chemical graphs.

This contribution is organized as follows: After introducing some basic graph-theoretic notation and discussing properties of chemical graphs, we give a formal account of the class of cut-and-join crossover operations. In particular, we show under which conditions degree-preserving and bond-order-preserving joins exist. We proceed by showing that under these conditions it is also always possible to construct crossover offspring that preserve graph-theoretical planarity of the structural formula. We then consider the problem of computing all recombinant offspring for a pair of parents for degree- or bond-order preserving operations. Continuing the analysis of the operators, we proceed to show that already in the degree-preserving case, cut-and-join crossover is sufficient to generate any molecular graph in a small population starting from a small molecular base set. In the empirical part of this work, we demonstrate that cut-and-join crossover produced highly diverse offspring that are nevertheless chemically plausible, synthesizable, and even drug-like with a sufficiently high probability that unrealistic proposal can be easily removed by well-established filtering steps. As a showcase application for cut-and-join crossover we describe its integration with REvoLd to search for ligands binding to four different target proteins. Finally, we close with a discussion of the main results and some open questions.

## Preliminaries

An undirected graph $G=(V,E)$ consists of a set of vertices *V* and a set of edges *E* consisting of unordered pairs of vertices. In a directed graph, *E* is considered as a set of ordered vertex pairs. Below, it will be convenient for simplicity of the presentation to focus on undirected graphs. Undirected graphs are equivalent to symmetric directed graphs, i.e., $xy\in E(G)\iff yx\in E(G)$, where *xy* and *yx* denotes directed edges between the vertices $x,y\in V$. Where necessary, we will write *V*(*G*) and *E*(*G*) to refer to the vertex and edge sets of *G*. We define the edge neighborhood of a vertex $N^G_e(v)=\{vw \mid w\in V(G), vw \in E(G\}$. In general we consider graphs with vertex and edge labels $\ell _V:V(G)\rightarrow L_V, \ell _E:E(G)\rightarrow L_E$, where $L_V$ and $L_E$ are finite sets of labels. In applications to chemistry, vertex labels designate atom types (most commonly chemical element, isotope, charge) and edge labels identify bond types.

A graph *H* is a subgraph of *G*, in symbols $H\subseteq G$, if *H* is a graph, $V(H)\subseteq V(G)$, and $E(H)\subseteq E(G)$. The subgraph *H* is induced by a vertex set $W{:=}V(H)\subseteq V(G)$ if $xy\in E(G)$ and $x,y\in W$ implies $xy\in E(H)$. For labeled graphs, furthermore, vertex and edge labels are inherited. A path in *G* is a sequence of pairwise distinct vertices $x_0,x_1,\dots ,x_n$ such that $x_{i-1}x_i\in E(G)$. A cycle $(x_1,\dots ,x_n)$ consists of the path $x_1,\dots , x_n$ together with the edge $x_nx_1\in E(G)$. A connected component is defined as a maximal connected subgraph $H = (V',E')$ of a graph *G*, where $V' \subseteq V$ and $E' \subseteq E$, such that there exists a path in *H* between *u* and *v*, for every pair of vertices *u*, *v* in $V'$.

Consider a bipartition $V=V'\cup V''$ where $V'\ne \emptyset$ and $V''\ne \emptyset$ and $V'\cap V''=\emptyset$ and denote by $H'=G[V']$ and $H''=G[V'']$ the two subgraphs of *G* induced by $V'$ and $V''$. The edge set ${{\,\textrm{C}\,}}(V',V''){:=}\{xy\in E(G)|x\in V', y\in V''\}$ is called the *cut set* of the cut $(V',V'')$. Then $E(H')$, $E(H'')$, ${{\,\textrm{C}\,}}(V',V'')$, and ${{\,\textrm{C}\,}}(V'',V')$ are pairwise disjoint and form a partition of *E*(*G*). Note that in the undirected case, ${{\,\textrm{C}\,}}(V',V'')={{\,\textrm{C}\,}}(V'',V')$ since symmetric edges *xy* and *yx* are identified in undirected graphs. A cut-set that is inclusion minimal, i.e., that does not contain any other cut-set as a proper cut-set is also called a *bond* in the graph-theory literature. We will not use this term here to avoid confusions with chemical bonds. By slight abuse of notation we write $V(E) {:=}\{x \mid xy\in E\} \cup \{y \mid xy\in E \}$ for the set of nodes incident with an edge-set *E*, such as a cut. The removal of the set of edges in a cut ${{\,\textrm{C}\,}}(V',V'')$ leads to the formation of two or more connected components. We say a cut is a *k*-cut, where *k* is the number of connected components induced by the cut.

**Fig. 1:**
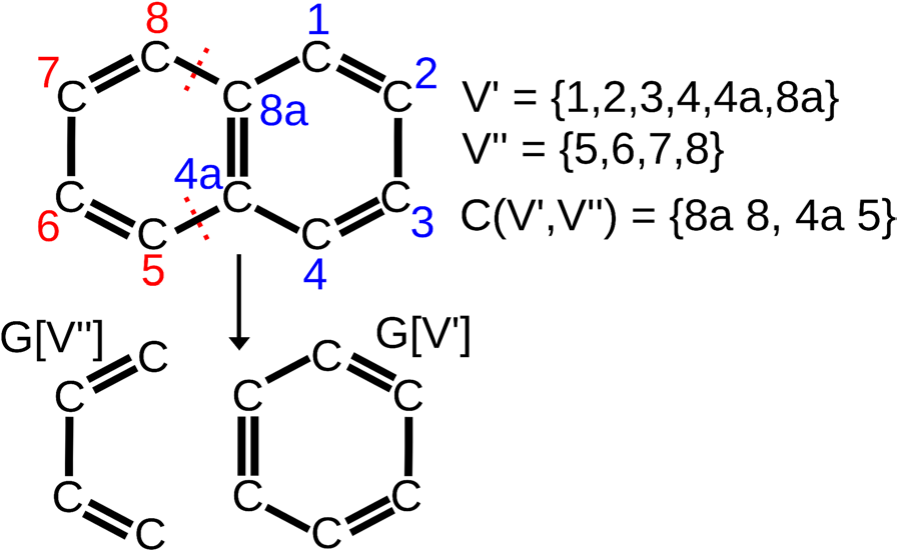
An example of a *2*-cut on naphthalene. The application of the cut set $C(V',V'')$ leads to the formation of the shown fragments, i.e the induced subgraphs $G[V']$ and $G[V'']$

In order to accommodate bond orders we allow multi-edges. The degree of a vertex $x\in V(G)$, denoted by $\deg _G(x)$, is then simply the number of incident edges, i.e., the multiplicity of edges is accounted for in the degree. For ease of presentation we will consider undirected, i.e., symmetric graphs throughout the main text. The introduced notation is illustrated in Fig. 1. Extensions to the directed setting are outlined briefly in the Appendix.

## Chemical graphs

**Fig. 2:**
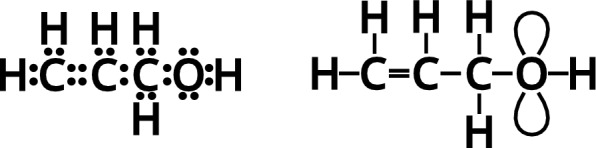
Lewis structure (left) and molecule multigraph (right) of allyl alcohol

The term *chemical graphs* is often used for labeled undirected graphs that represent chemical structural formulas. Here, we will use it more specifically to refer to the multigraphs that are equivalent to *Lewis formulas* [26] describing neutral compounds without unpaired electrons. In this setting, each bonding electron pair is represented as an edge, and each non-bonding electron pair as a loop. The bond order is therefore determined by the number of parallel edges. The electron pair of a bond is considered to be divided up between the two atoms that it connects, while a non-bonding pair is localized at a single atom. The number of electrons in the outer shell is usually preserved (at least for the atoms typically appearing in organic chemistry). The atom type therefore defines the degree of a vertex in the multigraph, which is given as the number of incident edges (counting multiplicities) plus twice the number of loops, see Fig. 2. This standard notion of degree in multigraphs agrees with Frankland’s “atomicity” and conforms to the IUPAC term *valency* [27]. We note in passing that the formalism can be extended in principle to accommodate unpaired electrons and charges e.g. by considering graphs with semi-edges [28]. Here, we will not make use of such extensions, however.

The oxidation state of an atom is implicitly determined by the different number of loops and bonds and thus can be changed by converting loops to bonds or *vice versa*. For example sulfur may have degree two like oxygen, in which case there are two loops. These may be exchanged for bonding electron pairs, as e.g. in $\hbox {SO}_3$. For our purposes we disregard oxidation states and thus assume that the number of loops, i.e., non-bonding electron pairs, is also fixed for a given atom type. This amounts to considering loop-free graphs where the degrees are fixed by the atom types.1It is not difficult to accommodate also changes in oxidation states by allowing loops to be removed or two cut-edges to be combined to a loop. For ease of presentation, we will not consider this issue further.

Instead of considering multigraphs explicitly, one can also use simple graphs with integer edge weights representing the multiplicity of bonds. We shall see below that these two representation give rise to different crossover operators, both of which are arguable useful for searching chemical spaces. From an algorithmic point of view, it is typically easier to work with edge-weights on simple graphs.

Even within the setting of edge-weighted labeled graphs that are equivalent to Lewis formulas there is no formal definition which graphs constitute viable molecules. 1 H-azirene, for instance, is a perfectly ordinary graph, and the molecule it represents even has a chemical name. Nevertheless, 1 H-azirene does not seem to exist. We therefore have to be consistent with operating on a space of labeled graphs that have an interpretation in chemistry. Moreover, the relationship of chemical graphs and molecules is not injective in general: tautomerism and mesomerism may lead to distinct graphs that represent the same molecule. These ambiguities are well known in the chemistry literature [29, 30]. They have very little impact on GA-based search, since the number of redundant representations usually is very small and the fitness evaluation can be performed for each of them independently.

Additional graph-theoretic constraints may be of interest to further restrict the admissible class of graphs. One such property is planarity. Since almost all molecules have planar structural formulas, it may be of interest to restrict the search space in this manner to rule out molecules with too many geometric constraints. At least some of these can be captured at least approximately in terms of graph-theoretic properties, such as the number of small rings or excessively high connectivity. Many topological filter criteria have been proposed in an effort to generate a large database of potential organic molecules [31].

### Mutation operations on graph genetic algorithms

A diverse collection of edit operations on graphs have been used in the literature, see e.g. [32, 33]. They share the property that they introduce small, local changes into a graph. Thus they can immediately serve as mutation operators in a GA. A non-exhaustive lists can be found in the Appendix. There are, however, several issues with these standard operations when applied to molecular graphs: Some of these operators (including vertex addition, node splitting, or edge deletion) do not preserve connectedness. It is of course possible to restrict these operations such that only connected graphs are produced. For example, node-splitting could be restricted to vertices that are not cut-vertices. Nevertheless, these operations are not readily applicable to chemical graphs, because in general they are not degree preserving. The only exception is node splitting of a vertex with even degree, where one could use operations of the form R-O-R to R-O-O-R and C to C=C such that each of the two carbons inherits two of the original carbons’ substituents.

A natural degree preserving mutation operator consists in a choice of two edges $e=xy$ and $f=uv$, cut both edges, and re-attaches the vertices as $e'=xu$ and $f'=yv$. Clearly, vertex degree is preserved. The operator can also be used as a way to model simple elimination reactions, since $\hbox {CH-CCl}$ can yield $\hbox {C}=\hbox {C}$ plus $\text {HCl}$. However, in cases where the resulting molecule is disconnected, the reattachment of edges necessarily produces isomers, i.e., does not change the chemical composition. This kind of *edge-swap* operation was recently used in models for the evolution of population structures [34].

## Cut-and-Join crossover operations on graphs

### Joins from cuts

Throughout this section we assume that we are given two labeled, connected, undirected graphs $G=(V,E)$ and $H=(W,F)$ as well as proper subsets $\emptyset \subsetneq V'\subsetneq V$ and $\emptyset \subsetneq W'\subsetneq W$ of their vertex sets. Writing $V''{:=}V{\setminus } V'$ and $W''{:=}W{\setminus } W'$, moreover it seems natural to associate the newly inserted edges with the edge cuts ${{\,\textrm{C}\,}}(V',V'')$, ${{\,\textrm{C}\,}}(V'',V')$, ${{\,\textrm{C}\,}}(W',W'')$, and ${{\,\textrm{C}\,}}(W'',W')$ that separate $G[V']$ from $G[V'']$ and $H[W']$ from $H[W'']$, respectively.

**Fig. 3:**
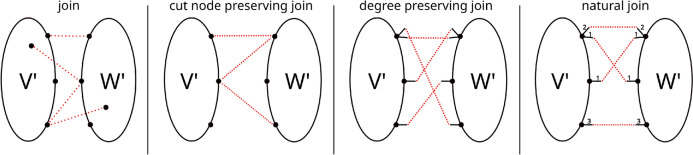
Increasingly restrictive join operators. While a join allows any nodes of two subgraphs to be linked, for a cut node preserving join only connections between nodes involved in the cut sets of their respective parental graphs are permitted. A degree preserving join further requires all nodes of the offspring graph to adhere to the degree of their respective parental vertices. The most restrictive join presented is the natural join, which introduces edge weights, e.g. bond orders, that must be conserved

We say that a cut $D={{\,\textrm{C}\,}}(V',V'')$ in a connected graph *G* is a *k*-cut if removal of the edge set *D* leaves *k* connected components. In particular, it is a 2-cut if both $G[V']$ and $G[V'']$ are connected (Fig. 3).

A natural construction for crossover is then to consider an “offspring graph” to be obtained by joining the induced subgraphs $G[V']$ and $H[W']$ by a set of edges connecting $V'$ and $W'$. More formally:

#### Definition 1

A graph *K* is a the result of a join of $G[V']$ and $H[W']$ if $V(K)=V'\cup W'$, $K[V']=G[V']$, $K[W']=G[W']$ and $E(K)=E(G[V'])\cup E(H[W'])\cup B_{\circ }$ where $B_{\circ }\subseteq V'\times W'$.

In its full generality, these joins encompass the multipoint graph crossover [16, 18, 20] as well as crossovers based on *induced* subgraphs. The subgraph crossover described in [16], however, is in general not of this type because there edges *within* a connected component can also be removed. This is not allowed in the cut-based operators described here. Depending on the application scenario, different constraints can be applied on how $G[V']$ and $H[W']$ are joined.

#### Definition 2

A join $K=G[V']\circ H[W']$ is called *cut-node preserving* if for each edge $xy\in B_{\circ }$ we have $x\in V'$, $y\in W'$, and there are edges $xx'\in {{\,\textrm{C}\,}}(V',V'')$ and $yy'\in {{\,\textrm{C}\,}}(W',W'')$.

In a cut-node preserving join, new edges are inserted only at vertices adjacent to the cut-edges in *G* and *H*, yielding a more stringent condition.

#### Definition 3

A join $K=G[V']\circ H[W']$ is *degree-preserving* if $\deg _K(x)=\deg _{G}(x)$ for all $x\in V'$ and $\deg _K(y)=\deg _{H}(y)$ for all $y\in W'$.

As an immediate consequence, degree-preserving joins can be constructed only from cuts of the same size. Conversely, cuts in *G* and *H* with the same number of edges can always be used to produce a degree-preserving join:

#### Lemma 1

If a join is *degree-preserving* is its also cut-node preserving.

#### Proof

If the join is not cut-node preserving, then there is a vertex *x* in $V(G)\cap V(K)$ or $V(H)\cap V(K)$ that is the endpoint of a join-edge while it is not incident to a cut-edge. Then $\deg _K(x)>\deg (x)$ in *G* or *H*, and thus the join is not degree-preserving. $\square$

#### Lemma 2

There is a degree-preserving join if and only if $|{{\,\textrm{C}\,}}(V',V'')] = |{{\,\textrm{C}\,}}(W',W'')|$.

#### Proof

Let $A{:=}{{\,\textrm{C}\,}}(V',V'')$ and $B{:=}{{\,\textrm{C}\,}}(V',V'')$ and $|A|=|B|$. Then there exists a bijection $\phi :A\rightarrow B$. Denote the cut edges in *G* by $xx'\in A$, and those in *H* by $yy'=\phi (xx')\in B$. Then the edges *xy* with $x\in V(G)$ and $y\in V(H)$ define a join. The $xx'\in {{\,\textrm{C}\,}}(V',V'')$ in *G* is replaced by the uniquely defined edge $xy\in E(K)$, we have $\deg _G(x)=\deg _K(x)$. Analogously, the edge $yy'\in {{\,\textrm{C}\,}}(W',W'')$ in *H* is replaced by the uniquely defined edge $yx\in E(K)$. Thus $\deg _G(x)=\deg _K(x)$ and $\deg _H(y)=\deg _K(y)$. Hence, the join is degree preserving. Now suppose $|A|\ne |B|$. Thus there is a vertex in $G[V']$ or $H[W']$ such that the degrees in *G* or *H*, and *K* respectively differ since there is a non-compensate join edge. $\square$

Lemma 2 yields a simple condition for the existence of a degree-preserving join: it is necessary and sufficient that the total weight of the cuts in *G* and *H* are the same.

Instead of considering multigraphs, one may also use integer edge-weights to describe bond orders. This gives rise to an even more restricted version of joins:

#### Definition 4

A join is *natural* if the number of edges with weight *h* is the same in *G* and *K* for all $x\in V'$ as well as in *H* and *K* for all $y\in W'$.

**Fig. 4:**
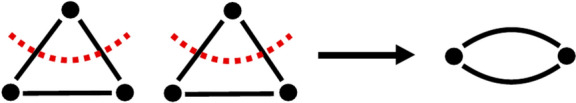
Choosing $V'$ and $W'$ as the top vertices of the two triangles yields two cuts consisting of two edges, each with weight 1. The only possible join in this case is a multigraph. This join is degree-preserving, as each of the two vertices still has two incident edges, but is not natural since it converted (parts of) a simple graph into a multigraph

In other words, a join is natural if the entire distribution of edge weights remains unchanged at each vertex. From a chemical perspective, this means that, for instance, a double bond must be “compensated” by a double bond in the recombinant graph *K* and cannot be “split” into two single bonds, or *vice versa*. Consider the example in Fig. 4:

The join of two nodes from triangles as shown in Fig. 4 is degree preserving but not natural since it changes two edges of weight 1 to a single edge of weight 2, and thus alters the distribution of edge weights at some vertices.

In contrast to the construction of degree-preserving joins, it is difficult in general both to determine whether a natural join exists and to construct and enumerate them. Essentially equivalent computational problems have been studied in the context of discrete tomography, a field concerned with the reconstruction of images from multiple projections. A short survey of pertinent results and references can be found in the Appendix. In brief, a linear-time algorithm exists to construct a natural join if there is only a single bond-type. In contrast, the generalization to two or more bond types becomes NP-hard even with various restrictions [35].

In the applications in later sections of this paper we are mainly interest in finding all (exponentially many) unique natural joins, which amounts to enumerating all possible recombinations of the parent fragments. In general, the number of unique solutions is exponential in the number *k* of bonds in the two cuts, as it can be well demonstrated on instances where each cut node is connected to a respective single cut edge of the same weight. With multiple bond types, the total number of candidate solutions is bounded by $\prod _{b} k_b!$, where $k_b!$ is the number of bonds of type *b* (e.g. single, double, triple, or aromatic). In principle one could simply generate all combinations of all permutations of the $k_b$ bonds of type *b* for the second partner of the join and check (in linear time) if the corresponding candidate join is indeed a valid natural join. In order to avoid the overhead of enumerating possibly large numbers of invalid candidates, we opted for an implementation as an integer linear programming (ILP) instance, which naturally lends itself for this purpose and yields a straightforward implementation. A detailed description of the ILP formulation, which is efficient and precise, including a proof of its correctness, can be found in the Appendix.

**Fig. 5:**
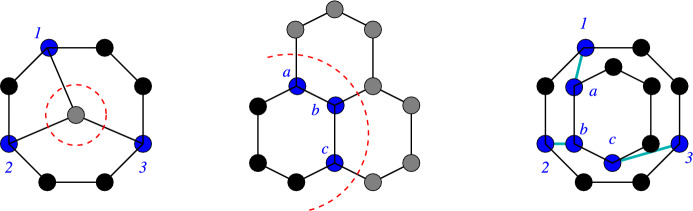
Cut-and-join crossover of two planar graphs can be restricted to yield again a planar graph. Since every minimal cut of a planar graph is cycle in its complement, there is a circular order to the severed edges and thus of the vertices incident with the cutset. It sufficies to preserve the circular order of these vertices (in arbitrary orientation). The example here is in addition degree-preserving

The appeal of cut-and-join crossover is reinforced by the following result on planar graphs.

#### Theorem 1

Let $G=(V,E)$ and $H=(W,F)$ be planar graphs and $A={{\,\textrm{C}\,}}(V',V'')$ and $B={{\,\textrm{C}\,}}(W',W'')$ be inclusion-minimal cuts in *G* and *H*, respectively, with $|A|=|B|$. Then there exists a planar join $G[V']\circ H[W']$. In particular, if $|A|=|B|$, then there is a planar degree-preserving join.

#### Proof

Since the edges of an inclusion-minimal cut in *G* correspond to a simple cycle in the planar dual $G^*$ of *G* [36], there is a planar embedding $G[V']$ such that all cut edges of $A={{\,\textrm{C}\,}}(V',V')$ point into “outside” of this cycle. This defines a circular order not only on *A* but also on the set $V^*$ of vertices incident to *A*. The same holds for $H[W']$, the cut edges ${{\,\textrm{C}\,}}(W',W'')$, and the vertices $W^*$ incident to this cut set. Thus there is an embedding of $G[V']$ and $H[W']$ such that $V^*$ lies on a geometric cycle $c_1$ and all other vertices in $V'$ are placed inside $c_1$, and similarly, $W^*$ lies on a cycle $c_2$ and all other vertices of $W'$ lie outside of $c_2$. Now, one can place $c_1$ inside $c_2$ and connect vertices in $V^*$ with vertices in $W^*$ such that these edges do not cross. Clearly the resulting join is planar. If $|A|=|B|$, there is a bijection of cut-edges that can be chosen to preserve the circular orders (in opposite directions). In particular, the $|A|=|B|$ new edges can be inserted to be degree-preserving: to this end, insert an edge between $x\in V^*$ and $y\in W^*$ and proceed clockwise along $c_1$ and $c_2$ to insert the desired number of (multi)-edges for *x* to *y* and then the clockwise neighbors of *y*. Then proceed to the clockwise neighbor of *x* and continue until all $|A|=|B|$ are inserted. By construction, no two edges between $V^*$ and $W^*$ cross each other, and thus the resulting embedding of the join is planar. $\square$

The construction of a planar degree-preserving cut-and-join crossover is illustrated in Fig. 5.

Since cuts of the same cardinality from planar structural formulas can always be recombined to yield a degree-preserving join that is again planar. The resulting graph has a high likelihood to be again a plausible structural formula. Moreover, planarity reduces the search space for finding a natural join as it restricts the density of bonds that can occur between multiple atoms.

Finally, we note that every cut-and-join crossover deriving from a 2-cut is reversible in following sense:

#### Lemma 3

Let $G=(V,E)$ and $H=(W,F)$ be connected graphs and $A={{\,\textrm{C}\,}}(V',V'')$ and $B={{\,\textrm{C}\,}}(W',W'')$ be inclusion-minimal cuts in *G* and *H*, respectively, with $|A|=|B|$, and consider the joins $G'=G[V']\circ H[W']$ and $G''=G[V'']\circ H[W'']$. Then (i) $G'$ and $G''$ are again connected and (ii) there is a cut-and-join crossover that produces *G* and *H* as cut-and-join crossover of $G'$ and $G''$.

#### Proof

Since we consider inclusion-minimal cuts, $G[V']$, $G[V'']$, $H[W']$ and $H[W'']$ are connected, und thus the joins are also connected. To see the reversibility consider the cuts ${{\,\textrm{C}\,}}(V',W')$ and ${{\,\textrm{C}\,}}(V'',W'')$ of $G'$ and $G''$, which recover $G[V']$, $H[W']$, $G[V'']$ and $H[W'']$ and note that ${{\,\textrm{C}\,}}(V',V'')$ in *G* and ${{\,\textrm{C}\,}}(W',W'')$ in *H* are legitimate joins for $G[V']$ and $G[V'']$, as well as $H[W']$ and $H[W'']$. $\square$

Clearly, this remains true for degree-preserving and natural 2-cuts.

The generation of recombinant graphs from two parent graphs *G* and *H* consists of two parts: (i) enumeration of a suitable set of cuts and (ii) construction of a set of recombinants. In the following two subsection we will consider these tasks in detail.

### Enumeration of cuts

A cut-set is redundant if it contains a strictly smaller cut $D'\subsetneq D$ as a subset.

#### Lemma 4

Suppose removal of the cut $D\subseteq E(G)$ decomposes *G* into three or more connected components. Then *D* is redundant.

#### Proof

Consider a cut $D\subseteq E(G)$ that partitions *V*(*G*) into three mutually disconnected vertex sets $V_1$, $V_2$, $V_3$. Note that $G[V_i]$ may itself be a disjoint union of connected components. For $\{i,j,k\}=\{1,2,3\}$ write $D_i{:=}\{xy| x\in V_j, y\in V_k\}$. Thus the edge sets $D_1$, $D_2$ and $D_3$ form a partition of *D*. If, say, $D_1=D_2=\emptyset$ then there is no edge between $V_3$ and $V_1\cup V_2$, and thus *G* was not connected to begin with. Thus at most one $D_i$ may be empty. If $D_1=\emptyset$, then $D=D_2\cup D_3$ comprises only the edges in $D_2$ between $V_1$ and $V_3$, and the edges in $D_3$ between $V_1$ and $V_2$, respectively. In particular, therefore, $D_2\subsetneq D$ and $D_3\subsetneq D$ are also cuts for *G*. If all three edge sets $D_i$ are non-empty, then each of $D_1\cup D_2$, $D_2\cup D_3$, and $D_1\cup D_3$ is a cut of *G* and a proper subset of *D*. Thus *D* is redundant because it can be obtained as a union of strictly smaller cuts. $\square$

Suppose we restrict ourselves to 2-cuts and sets of graphs without cycles, i.e. trees. Then every cut-set contains only a single edge and thus all possible degree-preserving joins are again trees. Similarly, if all graphs are trees or cacti, i.e., graphs in which every cycle forms its own block (2-connected component), then all 2-cuts either contain individual edges or cut a unique cycle in exactly 2 points. Any edge-preserving join therefore is again a cactus. A restriction to 2-cuts thus severely limits the generative power of degree-preserving crossover operators. We will have a close look at this issue below.

The problem of enumerating cut sets in a graph is a problem with a long history, see e.g. [37, 38]. It is well known that graphs may have an exponential number of minimum-weight cuts separating a given pair of vertices [39]. Algorithms for enumerating cuts thus are evaluated w.r.t. to the effort per cut. In [40] a deterministic algorithm with $O(|V|+|E|)$ delay between two output cuts has been described. Recently, an algorithm listing all inclusion minimal *s*, *t*-cuts in $\tilde{O}(|V|)$ amortized time (i.e., with an average running time linear up to logarithmic terms in |*V*| per cut) has been reported [41]. Surprisingly, computing the size of largest inclusion-minimal cut is NP complete [42].

**Fig. 6:**
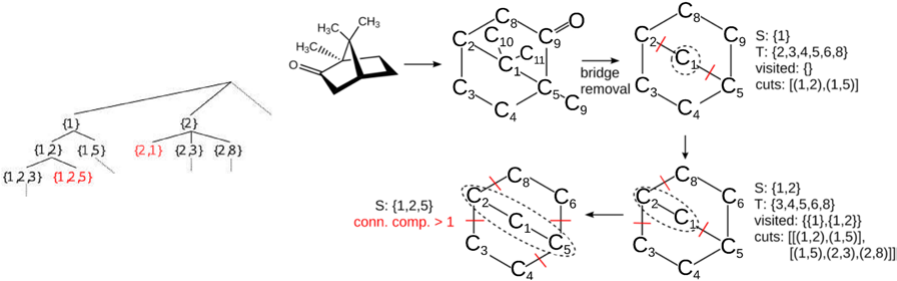
Left: Search-tree of the cut enumeration algorithm. Right: The different steps of the cut enumeration algorithm shown on camphor. Enumeration of a subtree can be halted, if a cut results in > 2 connected components or the cut node set has already been visited (colored red)

Since crossover at cut-sets of size 1 is rather trivial, we first extract the 2-edge connected components. These non-trivial scaffolds are then processed independently. For chemical graphs the simple algorithm outlined in Fig. 6 is used. For each initial vertex $x\in V(G)$ it starts from $V'=\{x\}$ and $V''=V\setminus V'$ and proceeds by moving a vertex $y\in V''$ that is adjacent to some vertex in $V'$ from $V''$ to $V'$ in each step. A cut-set is accepted if both $G[V']$ and $G[V'']$ are both connected. A branch in the search tree is abandoned if $G[V']$ or $G[V'']$ are not connected, or if the cut-set has been encountered already in another branch. In this manner we obtain all 2-cuts without being constrained to the inclusion-minimal cuts of *G*.

An interesting alternative to the enumeration of all cuts is to use a method of ordered generation that produces all cuts in the order of non-decreasing weights. The first method of this type was proposed in [43]. Later an improved version based on a different solutions of maximum flow problems was published [44].

### Natural joins as an ILP

Although it is possible to enumerate degree-preserving or natural joins directly, it is more convenient and elegant to rephrase these join operation as integer linear programming (ILP) problems. Standard ILP solvers (reviewed e.g. in [45]) can then be used to enumerate these joins. Let $C_G:= {{\,\textrm{C}\,}}(U',U'')$ for some graph *G* with weighting function $\ell _G$ and $C_H:= {{\,\textrm{C}\,}}(W',W'')$ for some graph *H* with weighting function $\ell _H$ such that $|C_G|=|C_H|$. Moreover, let the number of edges with weight *h* be the same for every $h\in {\mathbb {N}}$ in $C_G$ and $C_H$, hence there is a natural join. The goal is to generate all natural join edge sets $B_\circ$ to reconnect the connected components $U'$ and $W'$ derived from a cut. Let $e\in C_G$ and $f\in C_H$ and $u\in U',w\in W'$ such that *u* is incident with *e* and *w* is incident with *f*. We track whether to join *u* and *w* by an edge with weight $\ell (f)$ by variable $a_{ef}$ such that $a_{ef} = 1$ if *u* and *w* are joined by an edge, and $a_{ef}=0$ otherwise. This yields for the set of join edges $B_{\circ }=\{uw\ |\ a_{ef}=1\}$. For the edge-weights in the join graph $K=(U'\cup W', E')$ with $E':=E(G[U'])\cup E(H[W']) \cup B_\circ$ we have the labeling function $\ell _K(e)=\ell _G(e)$ if $e\in E(G)$ and $\ell _K(e)=\ell _H(e)$ else. For every cut-edge in $C_G$ and $C_H$, respectively, there is exactly one “joining edge” in $B_\circ$, which imposes the constraints

2 $$\begin{aligned} \sum _{e\in C_G} a_{ef} = 1 \text { for all } f\in C_H \quad {and}\quad \sum _{f\in C_H} a_{ef} = 1 \text { for all } e\in C_G \end{aligned}$$

The faithful preservation of edge-weights in *K* can be modeled by a simple constraint

3 $$\begin{aligned} \sum _{f\in C_H} a_{ef}\cdot (\ell _G(e)-\ell _H(f)) = 0, \text { for all } e\in C_G. \end{aligned}$$

Finally, we need to ensure that we do not introduce new multi-edges into the join graph *K*. For a vertex $u\in U'$, let $E_u$ annotate the edges of *u* that are incident to *u* in $C_G$ (analogously for $w\in W'$ and $E_w$). Since the introduction of multi-edges to *K* can only occur for vertices where $E_u$ and $E_w$, respectively, contain more than one edge, we obtain the following constraint:

4 $$\begin{aligned} \sum _{e\in E_u} \sum _{f\in E_w} a_{ef} \le 1 \quad \text {for all } u\in U', w\in W' \text { with } |E_u|,|E_w|>1. \end{aligned}$$

This constraint can be dropped whenever $E_u$ and $E_w$ do not contain any edges with the same weight, since then joining *u* and *w* in *K* is already ruled out by equation (3). Figure 16 provides a graphical representation of the relation between the constraints (2), (3), (4) and the different join operations. The natural joins are then obtained by maximizing the objective function

5 $$\begin{aligned} \sum _{e\in C_G}\sum _{f\in G_H} a_{ef} \rightarrow \max \end{aligned}$$

#### Theorem 2

The set $B_{\circ }=\{uw\ |\ a_{ef}=1\}$ is the set of join edges of a natural join if and only if the $a_{ef}$ are a solution of the ILP specified by equations (2) to (4).

The proof can be found in the appendix.

A major advantage of the ILP is that it can be modified easily to incorporate additional chemical constraints or optimizations to obtain natural joins between two molecular fragments. This can be achieved by either adding penalties or by excluding structure such as allenes $-\hbox {C}=\hbox {C}=\hbox {C}-$ by adding constraints on the neighborhoods in *G* and/or *H* for edges $e\in B_\circ$. In such a weighted version, ILP solvers can efficiently produce limited top-lists of candidates without the need for an exhaustive enumeration and subsequent ranking.

### Generative properties of degree-preserving joins

Crossover operators on strings are inherently limited in that they cannot introduce letters at positions where no such letter is present in the same position. More generally, crossover operators produce offsprings that are “between” the parents and thus more similar to their parents than the parents to each other [46]. This can lead to a loss of diversity that, in GA implementations, is counteracted by mutation operators designed to increase diversity.

**Fig. 7:**
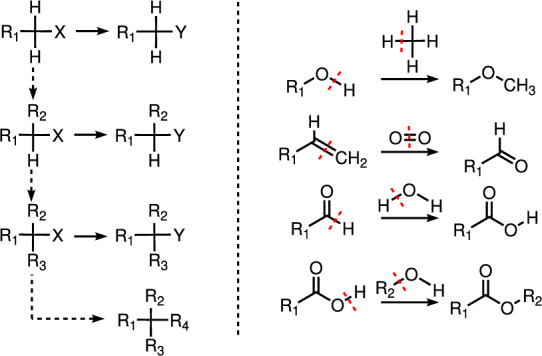
Left: Functional groups can be interconverted by a simple cut-and-join crossover. The general position (primary, secondary, tertary, quaternary) of a functional group can be changed by means of crossover operations that replace a hydrogen atom by a larger fragment (dotted arrows). Right: Cut-and-join crossover involving hydrogens enable access to simple reactions

Cut-and-join crossover in graphs unsurprisingly suffers from similar issues. Before we explore these limitations, however, we briefly discuss some of their desirable properties. Most importantly, functional groups can be easily transferred between molecular scaffolds. To this end, cuts of size 1 or 2 are sufficient, see Fig. 7. Labels, i.e., atom types, with the same valency can be replaced by cuts of size $\deg (x)$. Moreover, hydrogen atoms are readily exchanged for larger molecular fragments, provided there is a source graph from which the desired fragment can be cut. Simple reactions such as ether formation, esterification, etc., thus have their direct counterpart in cut-and-join crossover, see Fig. 7.

In a similar vein, it is easy to grow chains, e.g. by crossover of an H at the end of a chain with $\hbox {CH}_{4}$, if an extension by one carbon unit is desired, and to enlarge or shrink rings by cuts of size 2 and a subsequent join using two rings as parents. If the initial set of graphs ${\mathcal {G}}$ contains $\hbox {CH}_{4}$, $\hbox {NH}_{3}$, $\hbox {H}_{2}\hbox {O}$, and $\hbox {H}_{2}$, we can in particular build all cycle-free molecules even if only 2-cuts of size 1 are allowed. If only 2-cuts are allowed, however, cycles have to be present at the outset to allow the formation of cycles or additional bridges, since 2-cuts in cycle-free graphs necessarily have size 1. The closure of a new ring, however, requires that the join edges connected two vertices on one fragment with a vertex with degree-defect 2 on the other fragment, or two pairs of vertices.

**Fig. 8:**
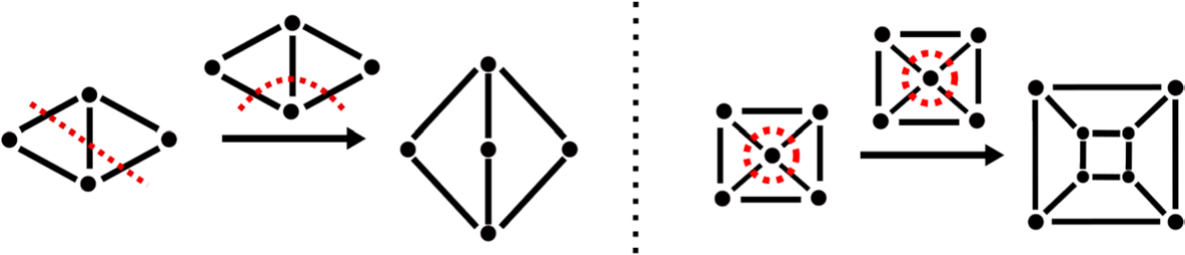
Within a minimal polycyclic set, bridged polycycles sharing multiple edges are accessible as well as cubanes

This can be achieved by also allowing 3-cuts of size 2, i.e., cutting two edges in *G* whose removal leads to three connected components. It is possible then to take two trees *G* and *H*, remove two disconnected pieces from both of them, and then form a (natural) join that introduces a cycle. The same operation also allows to convert $-\hbox {CH2}-$ to a carbonyl group (by crossover with water), to convert $-\hbox {CH2}-$ in a ring to a spiro-carbon by crossover with a 3-cut linear chain. Crossover of polycyclic compounds readily provides access to bridged compounds, see Fig. 8. These examples suggest that all molecular structures can be constructed by a sequences from degree-preserving crossovers from very simple building blocks.

This is true in a rather strong sense: for bounded degree graphs, a uniformly bounded size population and a number of crossover operations linear in the size of the target graph is indeed sufficient. In order to prove this statement formally, we need the following notation and terminology: Let *D* be a finite set of integers such that $1\in D$ and let ${\mathcal {S}}$ denote the search space comprising all loop-free connected graphs *G* such that all vertices $x\in V(G)$ have degree $\deg (x)\in D$. Moreover, we denote by ${\mathcal {T}}_D$ the set of star trees $S_k$ with a central vertex of degree $k\in D$. Note that for $k=1$, $S_1=K_2$, denotes the graph comprising two vertices connected with an edge.

#### Theorem 3

For every finite graph $G\in {\mathcal {S}}$ there is a sequence of of at most 2|*E*(*G*)| degree-preserving cut-and-join crossover and selection steps on a population of size $|{\mathcal {X}}|\le |D|+3$.

#### Proof

We proceed by induction in the number $n'$ of “heavy” vertices $x\in V(G)$ with degree $\deg (x)>1$. The statement is trivially true for all $G\in {\mathcal {T}}_0$ and these are by construction the only graphs with a single vertex of degree larger than 1. The only connected graph without a vertex of degree larger than 1 is $K_2\in {\mathcal {T}}_0$.

Now let *G* be a connected arbitrary graph and suppose the statement is true for all graphs with $n''<n'$ heavy vertices. First suppose that *G* contains a cut vertex *z*. Then consider the graphs $G_1$, $G_2$, ...$G_k$, $k\ge 2$, obtained by splitting *G* at the cut-vertex *z* such that (i) a copy of *z* is present in each $G_i$, and (ii) the vertex degree of *z* is restored by adding an edge and vertex of degree (i). Then *G* can ob obtained by recombining the $G_i$ sequentially, such that ${\tilde{G}}_i$ is composed of $G_1$ through $G_i$ for $1\le i\le k$. In particular, ${\tilde{G}}_1=G_1$. In each step, we cut *z* and its degree-1 neighbors from the rest of $G_i$, yielding a cut of size *h*. From ${\tilde{G}}_{i-1}$ we cut *h* degree 1 neighbors and then form the requisite join. By construction, ${\tilde{G}}_k=G_k$, and by induction hypothesis each of the $G_j$ can be constructed in the bounded population. To reach $G_k$, it thus suffices to keep ${\tilde{G}}_{i-1}$ in the population while constructing $G_i$. The number of cut-and-join steps equals the number of components and thus at most the number of edges incident to the cut vertex.

If *G* has a cut edge, between heavy vertices, there are two graphs $G_1$ and $G_2$ with strictly fewer heavy vertices, obtained by removing the cut edge and replacing the loose ends by vertices of degree 1. This process is easily reverted by a single cut-and-join crossover, requiring only $G_1$, $G_2$ in the population.

Finally, if *G* is biconnected, then we make use of the open ear decomposition [47] on the subgraph consisting of the heavy vertices only. If there is an ear with at least one interior (heavy) vertex, it suffices to construct the corresponding path by recombination of the growing chain with the star graph in a single step. Once the path is available it is recombined into *G*. If there are only ears left that consist of a single edge (i.e., that do not have interior vertices) between to heavy vertices, *uv*, then we consider the subgraph $G'$ from which *uv* is cut out (and loose ends are compensated by degree one vertices, and the fragment *uv*). Cutting the requisite number of degree 1 vertices from $G'$ and *uv*, respectively, there is a degree-preserving join that produced *G*. The insertion of an edge thus can be achieved with two cut-and-join steps: one for the formation of *uv* and one for the insertion of *uv*.

In summary, therefore, every graph $G\in {\mathcal {S}}$ can be produced by a number of cut-and-join crossover steps not exceeding twice the number of edges between heavy vertices starting from building blocks in ${\mathcal {T}}_0$. In each step we can produce the graph *G* by keeping in the population at most one graph $G^*$ with which the component under construction will eventually be recombined, the component $G_1$ currently under construction, a path *P* that will eventually be recombined with $G_1$ and the reservoir set ${\mathcal {T}}_0$ of building block that are required either to be composed into *P* or directly inserted into $G_1$ by recombination. Hence a population of size $|{\mathcal {T}}_0|+3$ is sufficient. $\square$

An analogous argument can be made for natural joins. This requires a larger set of building blocks, however, that contains “templates” for all multiple bonds, since these then cannot be obtained from cutting off a suitable number of degree 1 vertices but require cuts involving multi-edges for vertices with the same weight distribution of incident edges.

In practice, cuts that require cutting at both ends to a path may require up to $2 (D_{\max }-1)$ edges in the cut-set. It is convenient therefore, to use mutation operators that allow the insertion of an edge by means of edge swapping and the addition of a $K_2$ to be able to limit cuts to small sizes.

The construction in the proof of Thm. 3 has direct implications for the exploration of chemical space. First, it shows that functional groups can be introduced easily into a scaffold of interest. This can usually be achieved by a single cut of size 1 or 2 provided the functional group of interest is already appearing pre-formed in the population or provided as part of the reservoir set. This simple observation suggest to construct GAs for chemical space exploration in such a way that a reservoir set is always kept in the population as means of replenishing diversity that otherwise may be lost throughout a GA run: if an atom-type has been selected out of the population, crossover operators by definition are incapable of recovering it. It can be taken easily from the reservoir set however. In the field of retro-synthetic analysis, Stuart Warren defined a functional group interconversion (FGI) as “the process of converting one functional group into another by substitution, addition, elimination, oxidation, or reduction.” In our setting, all these operations can be achieved with degree-preserving cut-and-join crossover. For crossovers restricted to natural joins, however, the mutation operator is required to change bond order, or paths of length 2 need to be replaced by cuts with larger cut-sets.

**Fig. 9:**
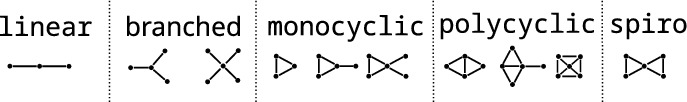
A minimal set of base graphs ${\mathcal {B}}$ needed to access all graphs of degree at most 4 using a sequence of natural join operators

It is also possible to restrict the cut operations to 2-cuts at the expense of a larger set of “base graphs” that provide building blocks to construct more complex scaffolds. We restrict the following discussion to graphs with degree at most 4, which in essence covers the search space of organic molecules. Starting point is the set of graphs ${\mathcal {B}}$ shown in Fig. 9.

We endow the set of all graphs ${\mathcal {G}}$ with a directed graph structure by inserting a directed edge (*G*, *H*) between two graphs $G{:=}(U,F)\in {\mathcal {G}}$ and $H=(W,D) \in {\mathcal {G}}$ if *H* is the result of a natural join of 2-cuts from *G* and one of the base elements $B_i\in {\mathcal {B}}$. Let us denote the resulting graph by $\vec {{\mathcal {G}}}$. In Additional file 1 we show in a purely graphical manner that all local neighborhoods of a vertex of degree at most four can be interconverted by natural cut-and-join crossover with members of ${\mathcal {B}}$. As an immediate consequence, we see that every graph can be stepwisely built up from the base set, and each of the construction steps is reversible by Lemma 3. This implies

#### Theorem 4

The search space graph $\hat{{\mathcal {G}}}$ is strongly connected.

We note that an analogous result can also be obtained for variants of graph construction in Thm. 3. To see this, it suffices to construct graphs containing the base elements and add them to the reservoir. As an alternative, it also suffices to consider 2-cuts and allow the opening or closing of a bond in exchange for a $K_2$, i.e., edge-swaps, as a mutation operator. The strong connectedness of the search space graph has important consequences for the performance of a GA built upon the cut-and-join cross-over operations: it ensures that any desired molecule can be reached with a non-zero probability from any starting point. This is true as long as there is a finite probability that the required base set or the repertoire set remains in the population for any finite number of steps. The reason is simply that the construction pathways described above are finite, and each step is realized with a finite probability.

## Evaluation and benchmarking of cut-and-join recombinants

### Diversity of recombinants

We first investigate to what extent natural or degree-preserving crossovers generates novelty. To this end, we use a small random seed set from the USPTO-10k “clean” dataset, a repository of patented molecules introduced in [48], and ask whether the crossover products are still contained in this data set. One starting molecule gives rise to 4,697 unique recombinants whereas five or ten yield 75,083 and 396,696, respectively (All used starting molecules are shown in Additional file 2). The test was performed by converting the crossover products to canonical InchIs [49], since they also account for resonance structures and tautomers, and comparing them. Only 3, 18, and 36 recombinants were recovered from the USPTO-10k database. Due to rdkit issues in converting molecular graphs, 30 InchI strings were invalid. Instead their respective canonical SMILES strings were used for further benchmarking.

**Fig. 10:**
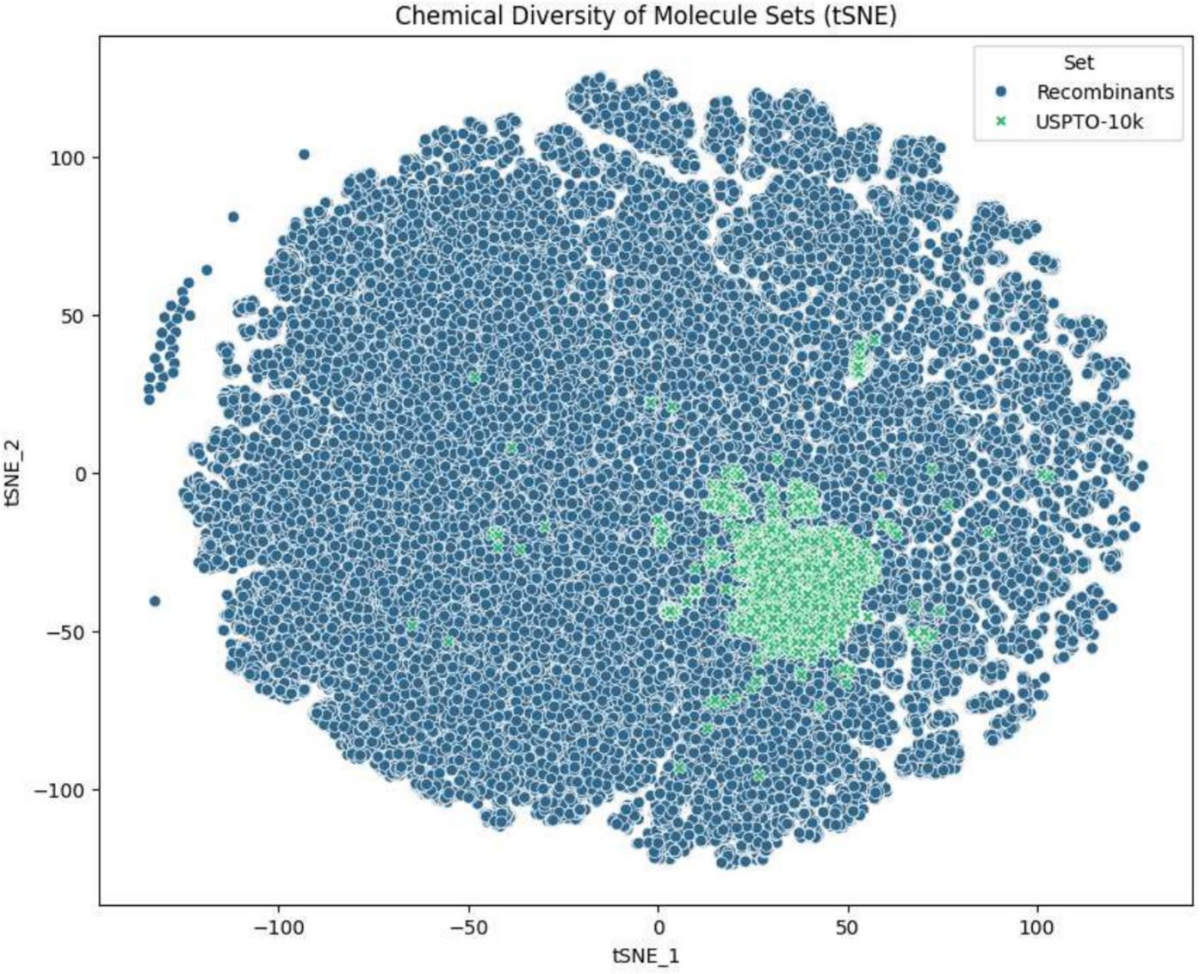
Diversity of crossover products taken from unrestricted cut-and-join operation on a sample of 10 molecules from the USPTO-10k. The homogeneous distribution of the recombinants in the t-SNE embedding shows that offspring molecules cover a high molecular diversity

In order to visualize the distribution of parent (USPTO-10k) molecules and crossover offspring molecules, we computed 1024-bit Extended-Connectivity Fingerprints (ECFP), a bit-vector representation of structural features in molecules including the circular substructures of each atom in a radius of 2 and performed a t-stochastic neighborhood embedding (tSNE) [50]. Figure 10 shows that the offsprings cover a much wider range also in terms of fingerprint descriptors.

### Post-processing of candidate molecules

While the restriction to possibly planar chemical graphs is an efficient pre-selection, many of these graphs will not represent stable, synthesizable molecules that are “drug-like”. Since cut-and-join can produce candidate molecules very efficiently it seems to be a useful strategy to prune the set of candidate graphs by removing chemically implausible entries. Commonly, molecular graphs are checked for *“molecular validity”* by means of two methods:

1. Parsing 1D string representations.
  For this either SMILES or SELFIES can be used. SELFIES have valency and bond configuration checks by design, so even random strings represent valid molecules. SMILES on the other hand can be sanitized, for example using RDKit. With the extension of SMARTS, substructure matches can be found and thus seemingly molecules containing instable functional groups can be removed.
2. 3D embeddability.
  A wide variety of methods have been devised to generate molecular geometries from structural formulas, see e.g. [51–54]. These can be used to the check the chemical plausibility of molecular graphs by generating a 3D model and checking whether interatomic distances and bond angles are within the ranges known from stable molecules.

We used a subset of the topological and functional group filters described in [31] and implemented them using SMARTS. The filters are divided into three tables. Filters regarding ring strain, such as small fused or spiro rings, eliminate 3, 108 molecules (0.8%). The other subset of filters is equipped for problems with unsaturated bonds, such as the filtering of difficult to synthesize allenes, effectively removing 55, 517 (14.0%) molecules. Functional group filters constitute the largest single category, excluding volatile and unaccessible functional groups such as peroxides or anhydrides. The majority, 128, 410 molecules (32.4%), were discarded by functional group filters.

The post-processing steps defined in Table 4 of [31] also included the conversion of functional groups to enrich chemical space. This was omitted since we aim to characterize the crossover products without further modification. Since rdkit had no suitable algorithm to properly classify bridgeheads in complex recombinant molecules, we abstained from including functions dedicated of identifying them. Moreover, we skipped checks of atomic volume. Instead, we directly considered 3D embedability using the rdkit implementation of basic knowledge informed Experimental-Torsion Distance Geometry (ETGK) [55]. The method attempts to place a molecule in 3D space such that known bond length, bond angles, and torsion angles for bonds are respected. If this is not possible, the violations are recorded.

We subjected 209, 665 filtered molecules of the 10 starting molecule recombinant set to the ETGK algorithm. In this set, 150, 175 molecular graphs (71.6%) were successfully embedded, while 28.4% showed at least one violation. Among the 59, 490 embedding errors, the most common issue is that planar arrangements of atoms (e.g. in aromatic systems) cannot be achieved in the minimization step of the embedding. In about a quarter of the non-embeddable molecules the method was unable to find an initial embedding. More details on the types of embedding violations can be found in Additional file 3.

### Synthesizeability

In real life applications, in particular in Drug Discovery, only molecules are of interest that are readily synthesizable. Many different measures of synthesizeablity have been proposed in the literature, see e.g. [56] for a recent overview. SAScorer, for instance, is pretrained on PubChem data and focusses on structural features. Penalties are given for large number of atoms, spiro centers, and bridges [57]. A lower score is associated with an easier access through synthesis. An alternative is to use SCScore [58], which is trained on reaction networks with the thought that synthetic complexity increases with the expected number of reaction steps. A higher score then indicates more steps required for synthesis.

**Fig. 11:**
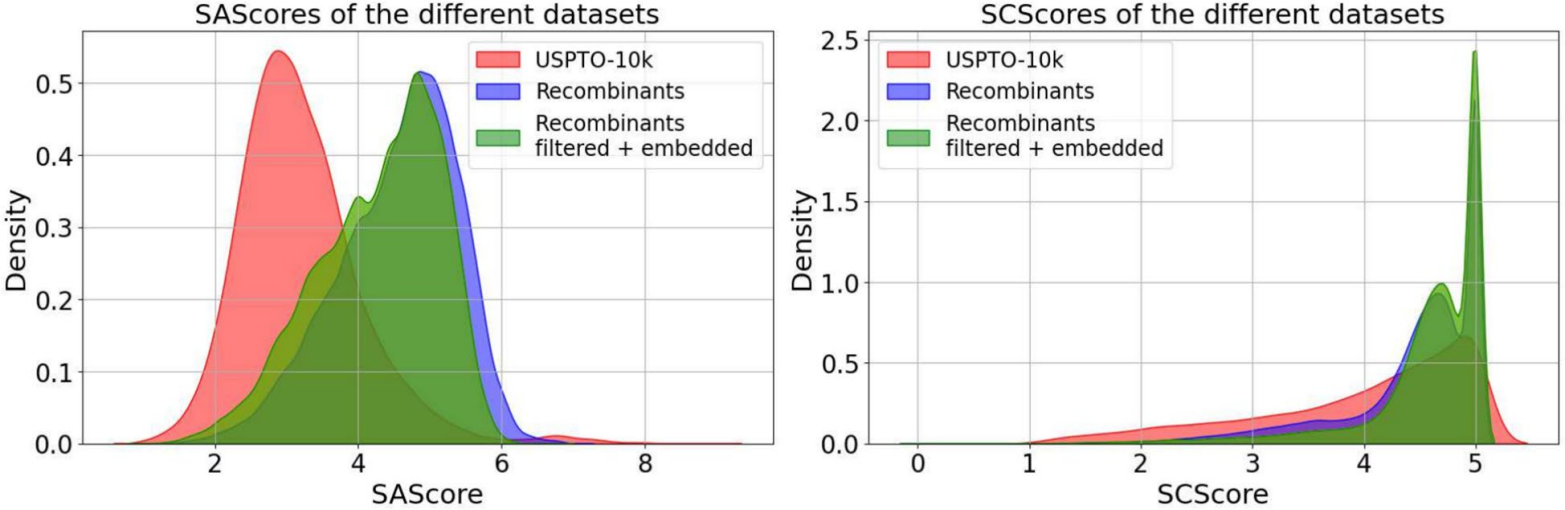
Synthesizability scores calculated by SAScore and SCScore on the 10-sample molecule recombinant set in comparison to the USPTO-10k dataset. Both tools show an increase in the difficulty of synthesizablility. The average $\Delta$SA Score is listed in Additional file 4

Distributions of synthesizability scores using both tools on the 10-sample molecule recombinant set are summarized in Fig. 11. As expected, we observed that the score distribution of crossover products is less favorable than the starting molecules. Nevertheless, the distributions show a large overlap and crossover products even with very good scores are not particularly rare. For the set of unfiltered recombinants, 60, 815 of 396, 700 molecules (15.3%) are located below the 75th percentile of the USPTO-10k SAScore (SAScore 3.63). In addition, the positive influence of the post-processing on the synthesizeablity is reflected by the fact that 35, 401 of 150, 175 (23.6%) of filtered and embedded recombinants exhibit an SAScore lower than the 75th percentile of the USPTO-10k.

**Fig. 12:**
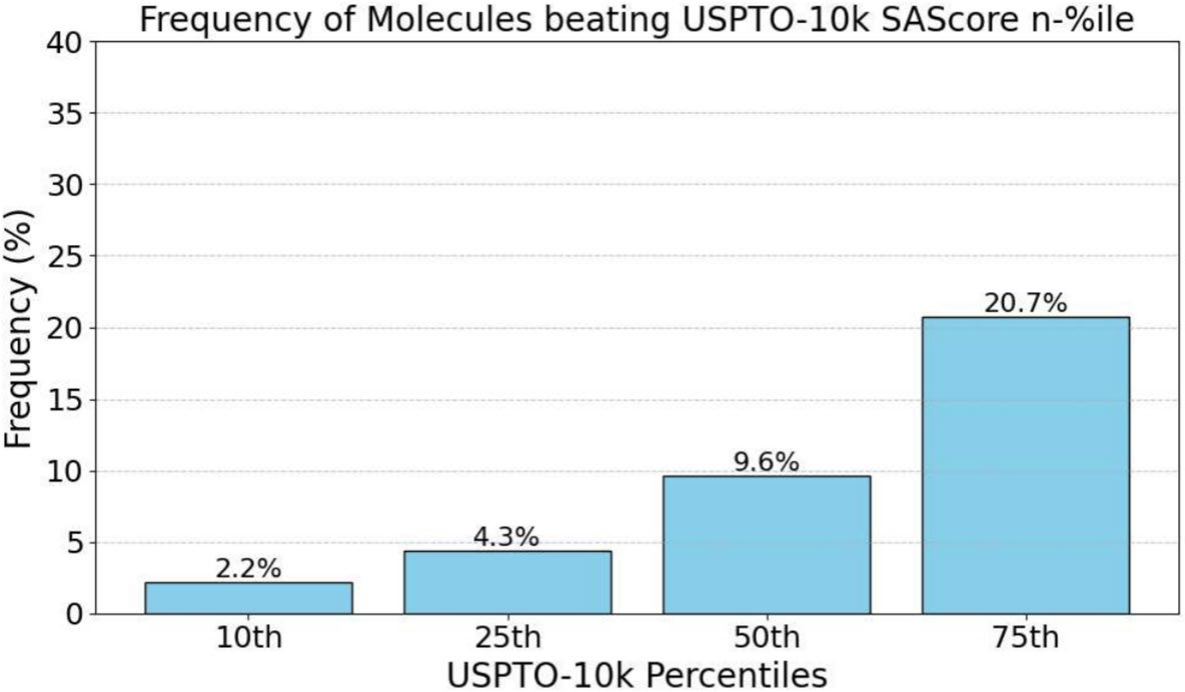
Frequencies of molecules with a better SAscore than the *n*th percentile of USPTO-10k molecules are depicted. A significant fraction of the recombinant offsprings has good SAscores, and thus it is feasible in each step of the GA to reject candidate offsprings that are difficult to produce according to their SA score

For the application in genetic algorithms, it of interest to analyze the efficiency of the presented method. Therefore, 150 randomly chosen pairs of unique parent molecules from the USPTO-10k dataset were subjected to the cut-and-join operation. Subsequently, offsprings were SA-scored and compared against the *n*th percentile of the reference dataset. Two pairs needed to be replaced, since the cut-and-join crossover works on the kekulized molecular graph, which timed out using rdkit (Fig. 12).

**Fig. 13:**
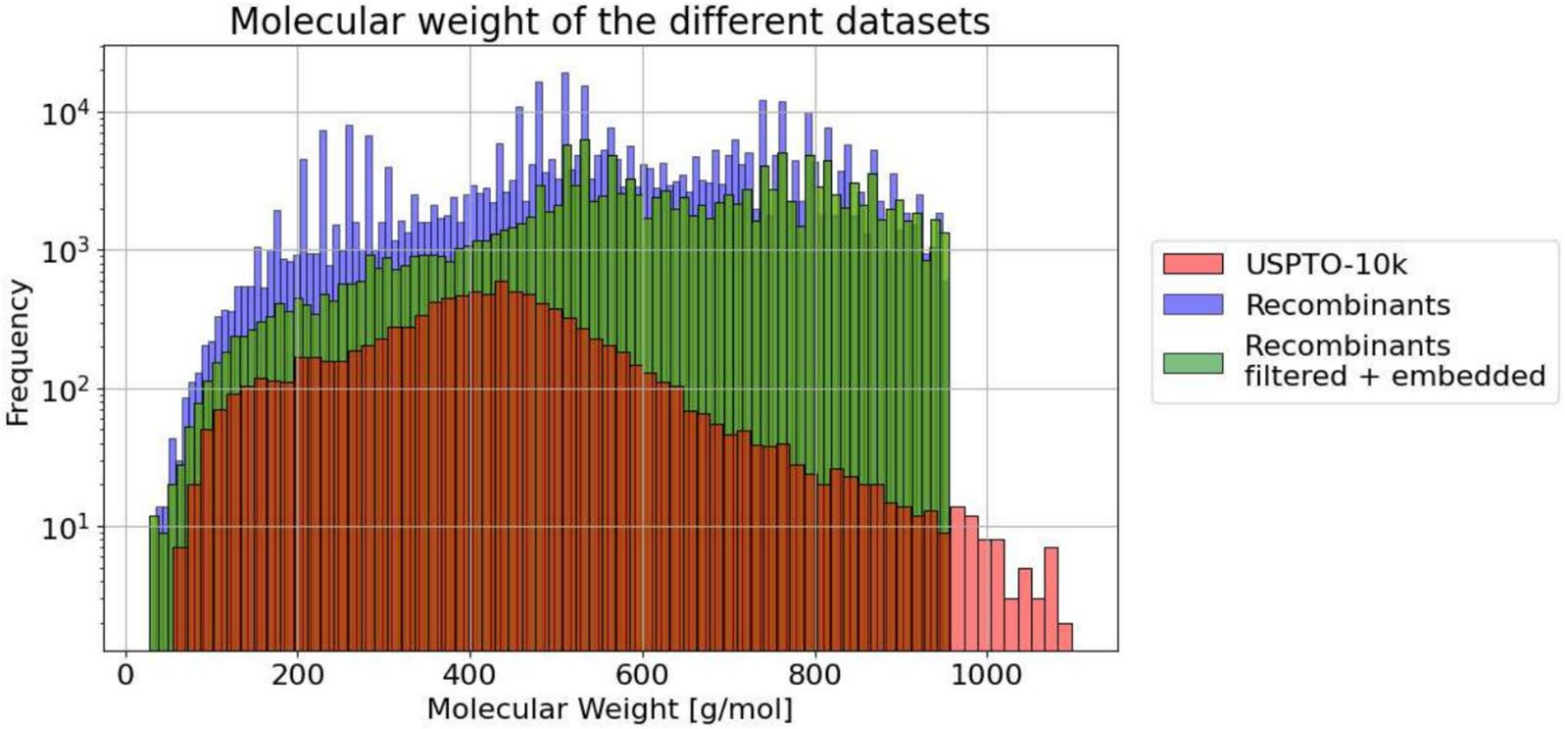
Molecular weight (MW) distribution of the 10 molecule sample set. The cut-and-join crossover operator shows an increase of molecular weight. The $\Delta$MW average of the different sets compared to the USPTO-10k can be found in Additional file 4. For better readability 56 molecules with a MW >1100 in the USPTO-10k were removed

It is feasible without dramatic performance loss, therefore, to reject candidates with large scores. Moreover, it may also be a viable strategy to only reject candidates derived from longer lineages of parents and grandparents with poor synthesizability scores, while allowing some search intermediates with unfavorable score to encourage the diversity (Fig. 13).

**Fig. 14:**
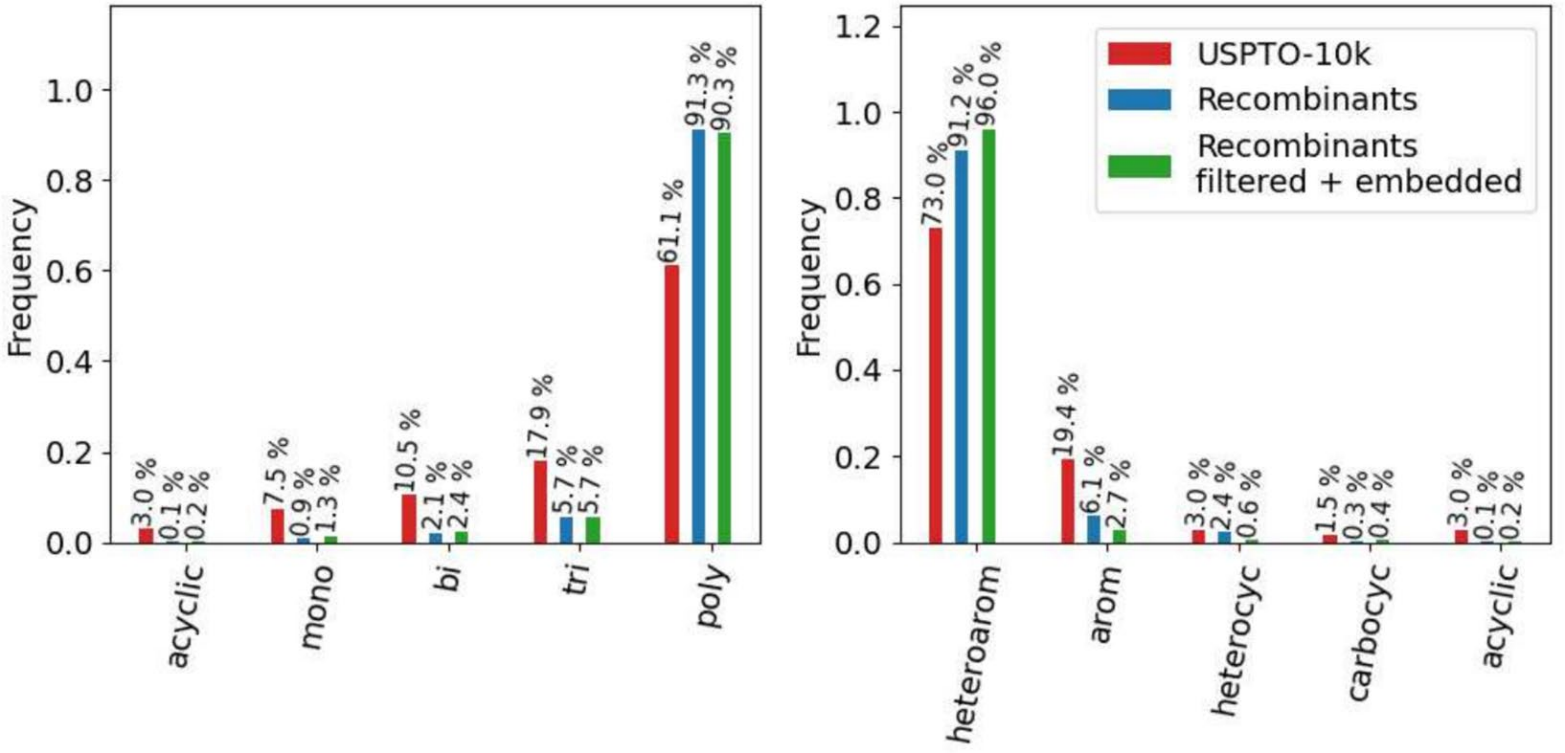
Distribution of ring topologies of parent and recombinant molecules. Molecules are assigned a category based on the priority: heteroaromatic > aromatic > heterocyclic > carbocyclic > acyclic. The cut-and-join crossover operator tends to generate more polycycles, especially with heteroaromatic and aromatic rings. This is also due to the sample, which is also largely composed of heteroaromatics

We also benchmarked the recombinant sets with the help of the MOSES platform. This tool is designed to support the evaluation of generative models for molecular structures. It computes an array of metrics for comparing molecular structures and properties interesting for drug discovery and compares the generated output to the characteristics of the data used to train the model [59]. Whenever possible 30, 000 molecules were sampled. As expected, all molecules passed the validity test based on valency and bond consistency. Checks for uniqueness were skipped since the cut-and-join algorithm is implemented in a way to not save duplicates. The novelty check, comparing canonicalized SMILES, confirmed that there is little to no overlap between the input database and the recombination products.

### Structural similarities of parents and recombinants

Molecule structures were further investigated by the cosine similarity in frequencies of Bemis-Murcko-Scaffolds (contracted molecule structures containing only ringsystems and linkers) [60] and BRICS-fragments (breaking of retro-synthetically interesting chemical substructures) [61].

Throughout all filter steps the cut-and-join operator gives very low Scaffold similarity of 1% to 5%, which reflects the ability to generate vastly different ring systems. In this application, the cut-and-join crossover operator leans towards the generation of polycyclic and heteroaromatic compounds as shown in Fig. 14, which is also caused by the sample being mostly made up of heteroaromatic compounds. The recombination products keep a balance of its BRICS fragments with similarities to the USPTO10k spanning from 19% to 45%.

Furthermore SNN (Similarity to a nearest neighbor) was calculated, which is the average between the Tanimoto similarity of ECFP fingerprints and its nearest neighbor from the USPTO-10k set. With similarities of 43% to 51%, generated molecules are neither suspiciously distant from the USPTO-10k nor very similar. With an Internal diversity score of 51% to 82%, the recombinant sets still shows uniqueness.

### Metrics for the application in drug discovery

Fréchet ChemNet Distance [62] is an evaluation metric for generative models that is particularly geared towards applications in drug design. It scores diversity and similarity in biological and chemical properties using the hidden layer of a neural network trained to predict biological activities. Products of the cut-and-join crossover tend to received rather high scores between 20.36 to 40.61 indicating the need to filter recombinants to enrich drug-like molecules. Similarly, the majority of recombinants score low in the Quantitative Estimation of Drug-likeness (QED) scale, which is based on molecular properties found in approved drugs. Nevertheless, 17% to 34% of the crossover products pass the medicinal chemistry filters (MCF) [59] and pan assay interference compounds (PAINS) filters [63].

The full table of MOSES metrics for the different recombinants can be found in Additional file 4.

In summary we observe that natural cut-and-join crossovers produce a large fraction of chemically meaningful candidates. Moreover, unrealistic candidates are easily removed using well-established filtering criteria. Even applying stringent filters for drug-likeness, a significant fraction of the crossover products are retained. It is important to note that in the benchmarking experiment reported here we only tested the suitability of the crossover procedure for chemical space exploration. It is perfectly acceptable in this setting that on average the offsprings are somewhat more difficult to synthesize and a moderate fraction does not conform to viable molecules at all. For successful applications of GAs to chemical structure search it is sufficient that in each step a significant fraction of good candidates are produced and that bad candidates can be rejected efficiently. Cut-and-join crossover for chemical graphs, i.e., structural formulae, clearly satisfies these criteria.

### Application to evolutionary optimization in computer-aided drug discovery

Evolutionary algorithms are typically very successful at generating and optimizing molecules, they share a common drawback—synthesizability [64–70]. To overcome this limitation RosettaEvolutionaryLigand (REvoLd) [71] was developed to exploit large chemical combinatorial libraries to increase synthetic accessibility and to reduce at the same time the number of dockings required to discover highly potent molecules. Despite very promising results, REvoLd is currently limited to predefined chemical spaces defined by make-on-demand libraries. These specifications are typically protected by commercial interests and only accessible through non-disclosure agreements. Moreover, these spaces usually encompass quite simple molecules and include mostly chemical matter of limited interest due to their enormous size.

**Fig. 15:**
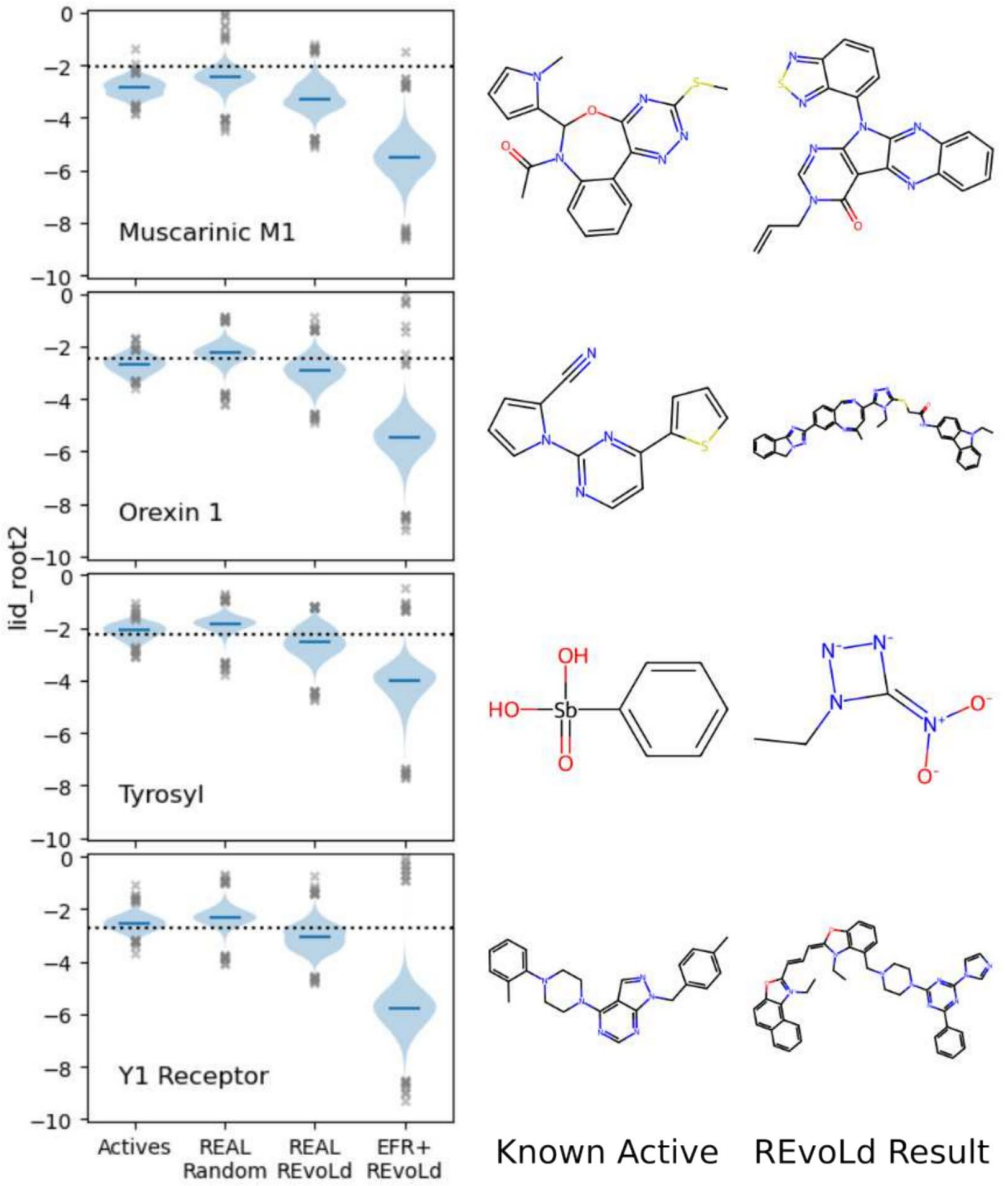
Distribution of normalized docking scores for four protein targets using either only known actives, random samples from the Enamine REAL space, REvoLd exploring the Enamine REAL space, or our custom chemical space. Dotted lines are docking scores for the co-crystallized ligands from the used protein structure. Next to each violin plot are first the highest scoring known active and second the highest scoring molecule from running REvoLd on our custom chemical space. Lid_root2 is the an interface score normalized by the squared number of non-hydrogen atoms. All scoring results except EFR+REvoLd were taken from the original REvoLd publication

To explore chemical space beyond this point and to demostrate the effectiveness of cut-and-join crossover, we used the natural crossover operator to generate and explore new combinatorial libraries from lists of known binders. We selected four of the five targets used as benchmarking targets in the original REvoLd publication [71]: the enzyme Tyrosyl and the receptors Orexin 1, Muscarinic M1, and Y1. The lists of known actives were taken from [72]. The screened libraries were generated from 292 (Tyrosyl), 234 (Orexin), 447 (Muscarinic), and 801 (Y1) known actives, yielding 481 million, 465 million, 182 million, and 316 million potential new molecules, respectively. 20 independent REvoLd runs were conducted against each target, all tested molecules reported, duplicates between runs removed, and the remaining molecules merged into a single results list per target. The results are presented in Fig. 15.

Our customized chemical space shows highly increased docking scores. REvoLd’s evolutionary optimization results in molecules exceeding the scores of previous results by far. The reported high scoring molecules show clear resemblance to the known actives used as seed molecules. In particular the resulting compound for the Orexin 1 receptor is promising, since it increased noticeably in size from known actives and thereby more closely resembles the large peptide that natively binds to the receptor. The compounds for Tyrosyl on the other hand are less convincing. They are more fragment-like and highly charged. However, the high scoring known actives used as seed molecules are small and polar as well, so this could be more a problem of the scoring function deployed for this target. We found the scoring function tends to overrate polar interaction and thus has a propensity to guide the evolutionary optimization towards even more charged compounds.

Overall, these simulation results are very promising, but prompt further investigation. The tremendously improved docking scores require rigorous testing to rule out possible docking artifacts. The case of Tyrosyl highlights target specific problems which require either a specialized scoring function or a different approach to generate the custom chemical libraries considering charges of resulting molecules. Furthermore, the synthetic accessibility needs a more thorough investigation, since this is a major bottleneck of evolutionary algorithms and generative methods in general in CADD. Finally, we noticed a ten times increased overlap within individual runs as well as between runs. This might point towards a smooth energetic landscape converging towards similar molecules, which would also partially explain the highly improved docking scores. Again, this also requires further investigation that, however, are outside the scope of this manuscript.

## Discussion and conclusions

We have introduced a very general scheme for defining crossover operators for graphs that start from cuts in the parental graphs and subsequently use a generalized join operation. This construction is very flexible and makes it possible to restrict the offspring to conserving certain properties of the parents. This makes cut-and-join crossover a generally applicable class of operators to implement recombination in genetic algorithms that operate on graphs. For applications to chemical graphs, degree-preservation and the preservation of graph-theoretical planarity are of particular relevance.

Cut-and-join crossover can be performed efficiently in such a way that degree preservation or the preservation of all bond orders can be enforced even though this is in general a hard problem. Moreover, alternative cuts can be enumerated efficiently in the order of increasing size.

In contrast to crossover on string representations, cut-and-join crossover on graphs is surprisingly versatile. In particular, we have shown that every finite graph with fixed maximal degree can be constructed by a GA operating on a finite size population within a finite number of steps. Moreover, the search space of such graphs is strongly connected. Taken together, therefore, the natural or degree-preserving cut-and-join crossover operations, possibly complemented by edge-swapping as a simple mutation operator, are sufficient to give access to any molecular scaffold. The natural and degree-preserving cut-and-join crossover conceptually resembles chemical reactions that exchange (large) moieties between pairs of molecules. Such exchanges appear to be particularly prevalent in metabolic networks. Our results on reachability and connectedness of chemical space under cut-and-join crossover thus suggests that metabolic reactions might also be capable of reaching all of chemical space. It is worth noting in this context that if a natural join $G[V'] \circ H[W'']$ exists, then there is also a natural join $G[V'']\circ H[W']$, which could be interpreted as a balanced reaction of the form

$$\begin{aligned} G + H \rightarrow \left( G[V']\circ H[W'']\right) + \left( G[V'']\circ H[W']\right) \end{aligned}$$

where $$V'\cup V''=V(G)$$ and $$W'\cup W''=V(H)$$.

Strong mathematical results on the convergence of GA require reachability together with non-trival assumptions on the selection procedure, and so far have become available only for strings [73, 74]. It will be interesting to explore whether cut-and-join crossover on graphs behaves similarly. This endeavor goes beyond the scope of the present contribution, however.

For chemical applications, crossover products that represent chemically plausible molecules are desired. Benchmarking our procedure using a wide variety of descriptors including 3D embeddability shows that a large fraction of the offspring of real-world molecular graphs satisfies these chemical constraints. Moreover, since alternative cut-and-join crossover products can be generated very efficiently, it is feasible to filter implausible graphs and/or graphs with other undesirable properties, so that GA runs can be restricted to well-formed molecules. This view is reinforced by the successful application of cut-and-join crossover to four drug-design tasks using the REvolD software. This showcase example led to predictions of candidate ligands with predicted binding constants substantially exceeding the best-known ligands. Of course, these computational candidates need follow-up with respect to their properties as drug designs, but in any case demonstrate the efficacy of cut-and-join crossover in a CADD setting.

The favorable properties of cut-and-join crossover operators suggest that such a general, mathematically motivated scheme is well applicable to chemical structure search. The method generates graphs that represent proper molecules with useful properties inherited from their parents at a sufficiently high density that there is no need for *a priori* restrictions of the crossovers. Instead mathematics-based generation with subsequent filtering for chemical sanity works efficiently.

Nevertheless, there are some aspects that deserve attention in future research. In particular, we have not investigated here how mutation and recombination operators interact. We expect that suitably designed mutation procedures can replace the reservoir sets of structural building blocks used as technical device in proving the reachability results. Moreover, *chirality* is an important feature of molecules that is not represented at the level of chemical (multi)graphs. These can thus be seen as representing the equivalence class of all stereo-isomers. In a brute-force manner, these can then be enumerated, see e.g. [75]. A more elegant approach would be to make use of annotation of stereochemical information which may be specified by structural formulas. *Cis/trans* isomers at double bonds may be specified in terms of the order of the substituents; the Natta notation, which uses wedges to indicate the spatial orientation in or out of the paper plane, is commonly used to describe tetrahedral chiral centers. Considering the neighborhood of a vertex as a circularly ordered instead of a plain set is sufficient to capture localized chirality [76]. It would thus be possible to handle chirality in the context of cut-and-join operations by inserting join edges into circularly ordered lists at the same places where the severed edges have been. Similarly, one could also deliberately invert chirality during the crossover operation. Moreover, the orientation of a chiral center is also a very natural mutation operation on molecular graphs with chirality annotation.

## Supplementary Information

Supplementary file 1. Proofs related to the strong Connectedness of the search space $\hat{{\mathcal {G}}}$

Supplementary file 2. Molecules used in the benchmarking section

Supplementary file 3. Details on embedding violations observed for crossover products

Supplementary file 4. Summary of MOSES statistics

## Figures and Tables

**Fig. 16:**
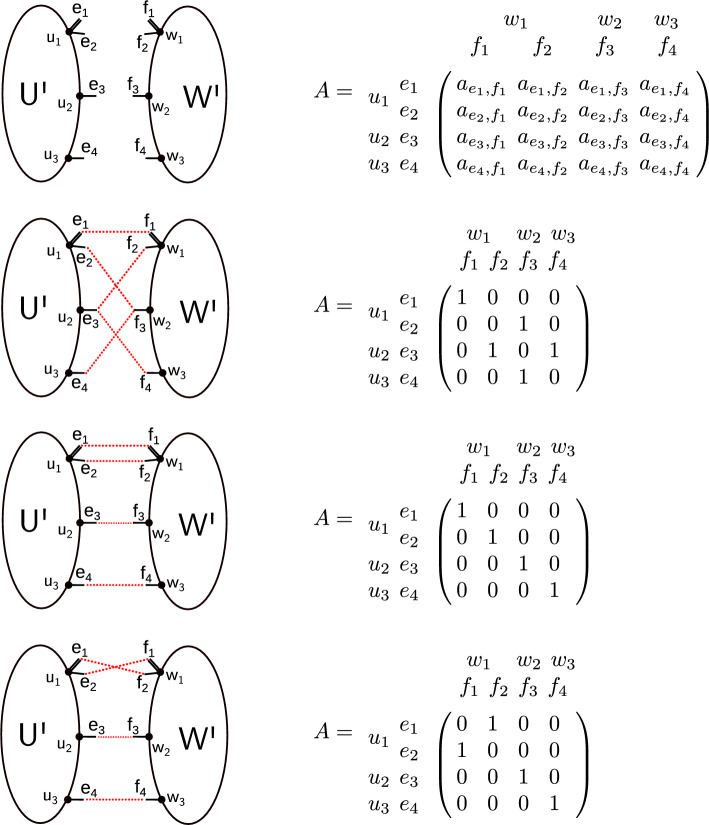
Illustration of $a_{ef}$ in matrix representation for different joins to demonstrate the necessity of the embedded constraints. The first two constraints only allow one connection per row and column, which is infringed in the first example by $e_3$ and $f_3$, which both give rise to two newly formed edges. The third constraint aligns the join to impede multi-graph formation, as it is depicted in the second example. The fourth constraint ensures that the edge labels of introduced edges adhere to the definition of a weight preserving join, violated in the forth example

## Data Availability

Data used in the study is available within the paper and its Supplementary Information files. Code for the cut-and-join operator is available under https://github.com/nicodomschke/cutandjoin. REvoLd is accessible under https://github.com/Hackefleisch/rosetta/tree/revold

## Mutation operators for graph GA

A wide variety of graph editing operations has been described in the literature. These operations can be readily used as mutation operators in GAs acting on populations of graphs. Most of these operations change vertex degrees, however, and thus are not directly applicable to the exploration of chemical spaces.

$$\begin{aligned} M_{vd}(G=(V,E))&= ( V \setminus \{v\}, E) \text {, random } v\in V \quad {\mathrm{(Vertex Deletion)}} \\ M_{va}(G=(V,E))&= ( V \cup \{v\}, E) \text {, random } v\notin V \quad {\mathrm{(Vertex Addition)}} \\ M_{cvd}(G=(V,E))&= ( V \cup \{w\}, E \cup \{vw\}) \text {, random } w\notin V, v\in V \quad {\mathrm{(Connected Vertex Addition)}}\\ M_{ed}(G=(V,E))&= ( V , E \setminus \{e\}) \text {, random } e\in E \quad {\mathrm{(Edge Deletion)}}\\ M_{ea}(G=(V,E))&= ( V , E \cup \{u,v\}) \text {, random } u,v\in V \quad {\mathrm{(Edge Addition)}}\\ M_{ec}(G=(V,E))&= ( V', E' )\text {, random } e\in E \quad {\mathrm{(Edge Contraction)}}\\ V'&= V \setminus \{e\} \cup \{w\} \\ E'&= \{f \in E:f\cap e = \emptyset \} \cup \{uw:\exists f\in E:u\in f\setminus e, f\cap e = \emptyset \} \\ M_{ns}(G=(V,E))&= ( V', E' ) \quad {\mathrm{(Node Splitting)}}, \\ \text {random }&v\in V, U_1 \cup U_2 = N(v), U_1 \cap U_2 = \emptyset \\ V'&= V \cup \{w\} \\ E'&= E \setminus \{uv : \forall u \in U_2\} \cup \{uw : \forall u \in U_2\} \\ M_{cns}(G=(V,E))&= ( V', E' ) \quad {\mathrm{(Connected Node Splitting)}}, \\ \text {random }&v\in V, U_1 \cup U_2 = N(v), U_1 \cap U_2 = \emptyset \\ V'&= V \cup \{w\} \\ E'&= E \setminus \{uv : \forall u \in U_2\} \cup \{uw : \forall u \in U_2\} \cup \{vw\} \end{aligned}$$

## Cut-and-join crossover for directed graphs

In the main text we considered only undirected graph for ease of presentation. The idea of cut-and-join crossover readily extends to directed graphs. The main results of this contributions also hold for this more general case.

In directed graphs, the order of the two subsets $V'$ and $V''$ of *V* matters: ${{\,\textrm{C}\,}}(V',V'')$ only contain the arcs (directed edges) with tail in $V'$ and head in $V''$. Considering undirected graphs as symmetric directed graphs, thus, an undirected cut set comprises the (disjoint) union ${{\,\textrm{C}\,}}(V',V'')\cup {{\,\textrm{C}\,}}(V'',V')$.

Degree-preserving joins for directed graphs need to consider in- and out-edges separately. That is, a degree-preserving join between $G[V']$ and $H[W'']$ exists if $|{{\,\textrm{C}\,}}_G(V',V'')|=|{{\,\textrm{C}\,}}_H(W',W'')|$ and $|{{\,\textrm{C}\,}}_G(V'',V')|=|{{\,\textrm{C}\,}}_H(W'',W')|$, i.e., if the number of severed outgoing arcs from $G[V']$ matches the number of severed incoming arcs in $H[W'']$ and *vice versa*. Natural join operations also need to consider in-coming and out-going arcs separately.

The results on reachability of graphs with bounded degree by means of degree-preserving crossover carry over to the directed case. The main difference is that larger, but still finite sets of base graphs are needed that cover all combinations of in- and out-degrees. Preservation of planarity is more difficult, however, since the relative order of in- and out-edges at each vertex incident to the cut set now matters.

## Recursive full enumeration of cuts

Since cuts are defined as proper bipartitions of the vertex set it suffices to enumerate all proper subsets $S\subset V(G)$. Since we are only interested in 2-cuts, we can restrict ourselves to connected subsets, i.e., connected induced subgraphs *G*[*S*] and $G[V\setminus S]$. We employ a branch and bound strategy: Starting for every node $v\in V(G)$ with the trivial initial cut $S = \{v\}$, we recursively add one new vertex to *S* at every step, with the restriction that we only may add nodes neighboring to any vertex already in *S*. If *G* is 2-connected, neither *S* nor *T* can never become disconnected using this scheme. We guarantee 2-connectedness prior to this step by removing all bridges as trivial cuts from the input graph and treating all resulting components as separate instances. A speed up of this procedure may be achieved by avoiding duplicate enumeration of subsets in different branches. For instance, the set $\{1,2\}$ may be reached by adding vertex 2 to $S=\{1\}$ or 1 to $S=\{2\}$. The order is irrelevant for both cuts and branch generation. We thus avoid recomputation by storing all already encountered sets *S* in a hash table and skipping any branch that already has been added to the hash table in a previous set.

## Existence of Natural Joins

The problem of constructing a natural join can be formalized as follows: For a set ${\mathcal {B}}$ of bond types and lists $V_1$ and $V_2$ of *m* and *n* atoms denote by $r_x^b$ and $s_y^b$ the numbers of cut bonds of atom $x\in V_1$ and $y\in V_2$, respectively. The task is to construct a bipartite graph such that each vertex $x\in V_1$ and $y\in V_2$ is incident with $r_x^b$ and $s_y^b$ bonds of each type $b\in {\mathcal {B}}$. Equivalently, this graph is represented by an $m\times n$ matrix *Q* with entries $q_{ij}\in {\mathcal {B}}\cup \{\varnothing \}$, where $q_{xy}=\varnothing$ denotes the absence of a bond. Clearly, $r_x^{\varnothing } {:=}m-\sum _{b\in {\mathcal {B}}} r_x^{b}$ and $s_y^{\varnothing } {:=}n-\sum _{b\in {\mathcal {B}}} r_x^{b}$. Moreover, we must have $\sum _{x} r_x^{b}=\sum _{y} r_y^{b}$ for each bond type *b*. For a fixed number of “colors” $k{:=}|{\mathcal {B}}|+1$, this problem is also known as the *k*-color tomography problem, *k*-CTP for short [35], as the $|{\mathcal {B}}|$-atoms consistency problem [77], and as the $|{\mathcal {B}}|$-color problem [78] in the context of structure reconstruction from discrete X-ray data. It is interesting to note in this context that the *k*-CTP can also be phrased as a maximal integer multiflow problem [79].

For $k=2$, i.e., a single bond type, and thus (0, 1)-matrices, the problem can be solved efficiently. It reduces to a variant of bipartite matching with an equivalent flow solution. More directly, it is solved by Ryser’s algorithm [80], which has a linear time complexity. The existence of a solution is determined by the Gale-Ryser theorem [81, 82], see also [83]. A compact formula to compute the exact number of such (0, 1)-matrices is given in [84].

A generalization of the Gale-Ryser theorem described in [85] allows to efficiently check if there exists a non-negative matrix with given row- and column-sum and entries bounded by a given positive integer. A generalization of Ryser’s algorithm for constructing such matrices is described in [86]. Nevertheless, if additionally the number of edges of color *b* is specified, the problem becomes NP-hard [87] when the size of the color set ${\mathcal {B}}$ remains unconstrained. Restricting the size of ${\mathcal {B}}$ by *k* is known as *k*-CTP which remains NP-hard for more than two colors, see [77] for $k\ge 7$, [78] for $k\ge 4$, and [35] for $k\ge 3$. Notably, the instances of *k*-CTP that appear in the context of cut-and-join crossover additionally limit the number bonds incident with each vertex. Although this exact constraint does not seem to have been studied, hardness results on highly restricted versions of the 3-CTP, reported in [88] strongly suggest that the *k*-CTP also remains hard for the chemically relevant cases of three or four bond types and row and column sums bounded by valency. Finally, it appears to be unknown if the problem becomes tractable if the colored bipartite graph defined by *Q* must be planar.

## Proof of correctness of the ILP formulation of natural joins

In the following, a graph resulting from a join operation specified by the ILP described in the main text and in appendix fig. 16 will be referred to as ILP-join-graph. More formally:

### Definition 5

(ILP-join-graph) Let $G=(U,E)$ and $H=(W,F)$ be weighted graphs with 2-cuts $C_G:=C(U',U'')$ and $C_H:=C(W',W'')$ and labeling functions $\ell _G,\ell _H$ as defined above. An ILP-join-graph of $G[U']$ and $H[W']$ is a join with $B_\circ :=\{uw | a_{ef} = 1\}$ and $a_{ef}$ determined by the ILP (2)-(5).

We first show that the ILP indeed produces natural joins:

### Lemma 5

Given two weighted graphs $G=(U,E)$ and $H=(W,F)$ with 2-cuts $C_G:=C(U',U'')$ and $C_H:=C(W',W'')$, the ILP-join-graph *K* that is obtained by maximizing (5) under constraints (2)-(4) is a natural join of $U'$ and $W'$.

### Proof

Let $G=(U,E)$ and $H=(W,F)$ be two weighted graphs with labeling functions $\ell _G$ and $\ell _H$. Let $C_G=C(U',U'')$ and $C_H=C(W',W'')$ be 2-cuts of *G* and *H* respectively and let *K* be the ILP-join-graph for $U',W',$ $C_G$, and $C_H$ with the introduced join-edges $B_\circ$. We first observe, that constraints in equ.(2) and the maximality w.r.t. to the objective function, equ.(5) always yield:

$$\begin{aligned} Z=\max \sum _{e\in C_G} \ \underbrace{\sum _{f\in C_H} a_{ef}}_{= 1, \forall e \in C_G}=\max \sum _{e\in C_G} 1 =|C_G| \end{aligned}$$

Analogously, we have:

$$\begin{aligned} Z=\max \sum _{f\in C_H} \ \underbrace{\sum _{e\in C_G} a_{ef}}_{= 1, \forall f \in C_H}=\max \sum _{f\in C_H} 1 =|C_H| \end{aligned}$$

By definition of $B_\circ$ it follows

$$\begin{aligned} |B_\circ | = \sum _{\{uw | a_{ef}=1\}} 1 = \sum _{e\in C_G} \sum _{f\in C_H} a_{ef} \end{aligned}$$

which is maximized under the objective function. Thus $|B_\circ | = Z$ and therefore $|C_G|=|C_H|=|B_\circ |$. With $\emptyset \subsetneq U' \subsetneq U$ and $\emptyset \subsetneq W' \subsetneq W$ we have $B_\circ \ne \emptyset$.

1. *K* is a cut-node preserving join: $B_\circ \ne \emptyset \overset{Def. K}{\Rightarrow }\ uw\in B_{\circ }\Leftrightarrow a_{ef}=1, e\in C_G, f\in C_H$ with *u* incident to *e* and *w* incident to *f*. We have $\ uu'=e, ww'=f$ since $E(K)=E(G[U'])\cup E(G[W']) \cup B_{\circ }$. Hence *K* is the result of a cut-node preserving join of $G[U']$ and $H[W']$.
2. *K* is a degree-preserving join: Since *K* is cut-node preserving, we only have to show that for all $u\in U'$ holds $deg_G(u)=deg_K(u)$ and for all $w\in W'$ holds $deg_H(w)=deg_K(w)$. Let $u\in U'$ and let $|E^G_u|, |E^K_u|$ be the number of edges incident to *u* in *G* and *K*. Let further $G^*:=(U, E{\setminus } C(U'U''))$, $K':=(U',E({G[}U'{]}))$ and $|E^{G^*}_u|$ be the number of edges that will not be changed due to the cut-and-join operation. Then clearly, $|E^{G^*}_u|=|E^{K'}_u|$. Hence, we only have to show that $|E_u^{C_G}|=|E_u^{B_\circ }|$. We compute:
  $$\begin{aligned} \left| E^{B_\circ }_u\right| =\underset{\{e=uu'| uu'\in C_G\}}{\sum }\ \underbrace{\underset{f\in C_H}{\sum }\ \ a_{ef}}_{=1, \ \forall e\in C_G}=\underset{\{e=uu'| uu'\in C_G\}}{\sum } 1 = \left| E^{C_G}_u\right| \end{aligned}$$
  An analogous argument can be made for each $w\in W'$. Thus *K* is degree-perserving.
3. *K* is a natural join: We observe $\ell _G(e) = \ell _K(e)$ for all $e\in E{\setminus } C_G$ and $\ell _H(f) = \ell _K(f)$ for all $f\in F{\setminus } C_H$, hence we only have to consider the weights of cut-edges. Constraint 1 implies, that for each $e:=uu'\in {C_G}$ there exists exactly one $f:=ww'\in C_H$ such that $a_{ef}=1$ and $uw\in B_\circ$. Analogously, for each $f:=ww'\in {C_H}$ there exists exactly one $e:=uu'\in {C_G}$ such that $a_{ef}=1$ and $uw\in B_\circ$. Hence, each cut-edge is substituted by exactly one join-edge. It remains to show that the introduced join-edge has the same weight as the edges it substitutes. The labeling of *uw* in the ILP-join-graph *K* is defined by the corresponging cut-edge in *H* as $\ell _K(uw):=\ell _H(ww')$. Constraint (3) yields:
  $$\begin{aligned} \begin{aligned} 0&=\sum _{f'\in C_H} a_{ef'}\cdot (\ell _G(e)-\ell _H(f') \overset{Con 1}{=}\ \ell _G(e)-\ell _H(f)\\ \Leftrightarrow \ell _G(e)&= \ell _H(f)= \ell _K(e) \end{aligned} \end{aligned}$$
  Thus *K* is indeed a natural join.

$$\square$$

In order to prove the converse we will need the following property of natural joins:

### Lemma 6

Let $C_G=C(U',U'')$ and $C_H=C(W',W'')$ be 2-cuts for two graphs $G=(U,F)$ and $H=(W,D)$ and let $K=(V,E)$ be a natural join of $G[U']$ and $H[W']$. There exist

$$\begin{aligned} g: C_G\rightarrow C_H, uu'\mapsto ww', \end{aligned}$$

bijective with $uw\in B_\circ$. In particular, we have $\ell _G(uu')=\ell _H(ww')=\ell _K(uw)$.

### Proof

Since *K* is a natural join, it is degree preserving and thereby also cut-node preserving. This implies that $|B_\circ | = |C_G|=|C_H|$ which, in consequence implies the existence of a bijection from $C_G$ to $C_H$. Let $E_u^{C_G, h}$ denote all edges of weight *h* in $C_G$ (resp. for $E_u^{C_H, h}$, $E_u^{B_\circ , h}$). First we observe for all $h\in {\mathbb {N}}$:

$$\begin{aligned} |E^{C_G,h}_u|= |E^{B_\circ ,h}_u|, \forall u\in U' \text { and } |E^{C_H,h}_w|=|E^{C_H}_w|, \forall w\in W'. \quad\quad (*) \end{aligned}$$

We define

$$\begin{aligned} f&: B_\circ \rightarrow C_G, uw\mapsto uu', \text { and}\\ h&: B_\circ \rightarrow C_H, uw\mapsto ww' \end{aligned}$$

recursively, as follows. Let $B^1_\circ : = B_\circ , C_G^1:=C_G, C_H^{1}:=C_H$. We choose in the *n*-th step $uw\in B^{n}_\circ$ at random and define $f(uw): = uu', h(uw)=ww'$ such that $\ell _K(uw)=\ell _G(uu') = \ell _H(ww')$, which is always possible by our previous observation $(*)$. Next, we let $B^{n+1}_\circ := B^{n}_\circ {\setminus } \{uw\}, C_G^{n+1}:=C_G^n{\setminus } \{uu'\}, C_H^{n+1}:=C_H^n{\setminus } \{ww'\}$ and proceed until $B_\circ ^{n+1}=C_G^{n+1}=C_H^{n+1}=\emptyset$. It is easy to see that *f* and *h* are bijective and we obtain:

$$\begin{aligned} g:C_G \rightarrow C_H, uu' \mapsto (h\circ f^{-1})(uu') = h(f^{-1}(uu')) \end{aligned}$$

which has the desired properties and by construction

$$\begin{aligned} \ell _G(uu')=\ell _H(g(uu'))=\ell _H(ww')=\ell _K(f^{-1}(uu')) = \ell _K(uw),\forall uu'\in C_G. \end{aligned}$$

$$\square$$

### Lemma 7

Let $C(U',U'')$ and $C(W',W'')$ be 2-cuts for two graphs *G* and *H* and let *K* be the natural join of $G[U']$ and $H[W']$. Then *K* can be obtained as ILP-join-graph from a solution of (5) under constraints (2) to (4).

### Proof

We set $a_{ef}=1$ for all $e\in C_G$ with $g(e)=f$ with *g* bijective as in Lemma 6. It remains to show that this assignment constitutes a solution to (5) under constraints (2) to (4).

(2): The bijectivity of *g* yields $\sum _{e\in C_G} a_{ef} = 1$ and $\sum _{f\in C_H} a_{ef} = 1$. Therefore, our construction of $a_{ef}$ satisfies (2).
(3): Since by Lemma 6$\ell _G(e)=\ell _H(g(e))=\ell _H(f)$, we have for all $e\in C_G$: $$\begin{aligned} 0&= \ell _G(e)-\ell _H(g(e)) \\&= \ell _G(e)-\ell _H(f)\\&= \sum _{f'\in C_H} a_{ef'}\cdot (\ell _G(e)-\ell _H(f')) \hspace{12pt} \text {(assignment } a_{ef}{).} \end{aligned}$$
(4): Assume there are $u\in U', w\in W'$ such that $\sum _{e\in E_u}\sum _{f \in E_w} a_{ef}>1$. This implies that there are two distinct edges $uw \in B_\circ$ which contradicts that *K* is a simple graph.
(5): Constraints (1) and (2) together with the objective function always yields $Z=\max \sum _{e\in C_G} \sum _{f\in C_H} a_{ef}=|C_G|=|C_H|$. Since *g* is bijective, and we construct $a_{ef} = 1 \iff g(e)=f$ we have for our constructed ILP-solution $|\{a_{ef}\ |\ a_{ef}=1\}|=|C_G|=Z$, hence our construction yields an optimal solution to (5).

$$\square$$

As an immediate consequence of Lemma 5 and Lemma 7 we obtain the following proposition:

### Proposition 5

Let $C(U',U'')$ and $C(W',W'')$ be 2-cuts for two graphs *G* and *H*. Then every possible natural-join of $G[U']$ and $H[W']$ can be obtained as an optimal solution of (5) under constraints (2) to (4).
