# Supplementary material for: Crossover operators for molecular graphs with an application to virtual drug screening
Source: J Cheminform. 2025 Jun 17;17:97. doi: 10.1186/s13321-025-00958-w (PMC12175394; doi:10.1186/s13321-025-00958-w)
Supplement: Supplementary file 1 — Proofs related to the strong Connectedness of the search space $\hat{{\mathcal {G}}}$G^ [file 13321_2025_958_MOESM1_ESM.pdf]

ADDITIONAL FILES

Additional file 1.

Strong Connectedness of the search space $\hat{\mathcal{G}}$

Theorem 6 (Strong-connectedness of the search space)**.** *The search space graph $\hat{G}$ is strongly connected.*

Proof. For every $G \in \mathcal{G}$, with $\hat{G} = (\mathcal{G}, E)$, there exists one of the following cuts:

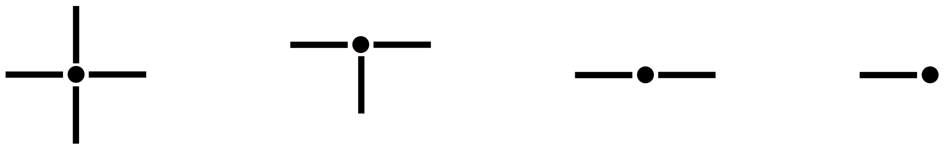

It is easy to see, that we can obtain each of these cuts from one of the base element $B_i \in \mathcal{B} \subseteq \mathcal{G}$. As a consequence, B_i can be reconstructed with the respective complement. Hence, for each $G \in \mathcal{G}$ there is a $(G, B_i) \in E$ for some $i \in \{1, \ldots, 10\}$. Therefore, it suffices to show, that base elements can be transformed into each other via a sequence of cut and join operations, in particular: $\forall B_k, B_l \in \mathcal{B}$ exists a path $(B_k, \ldots, B_l) : (B_i, B_j) \in E$ where all $B_i, B_j \in \mathcal{B}$. We construct:

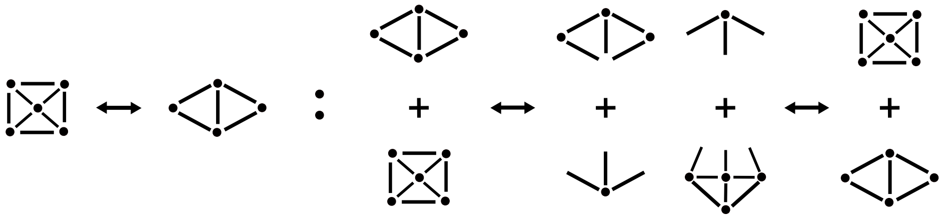

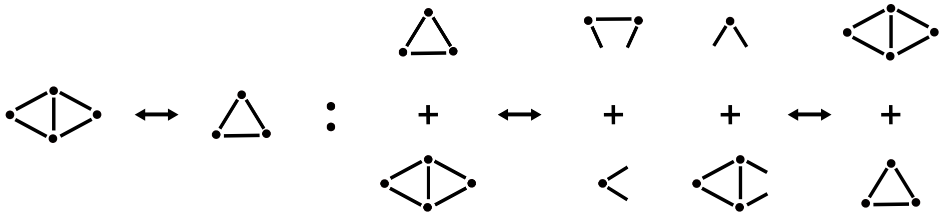

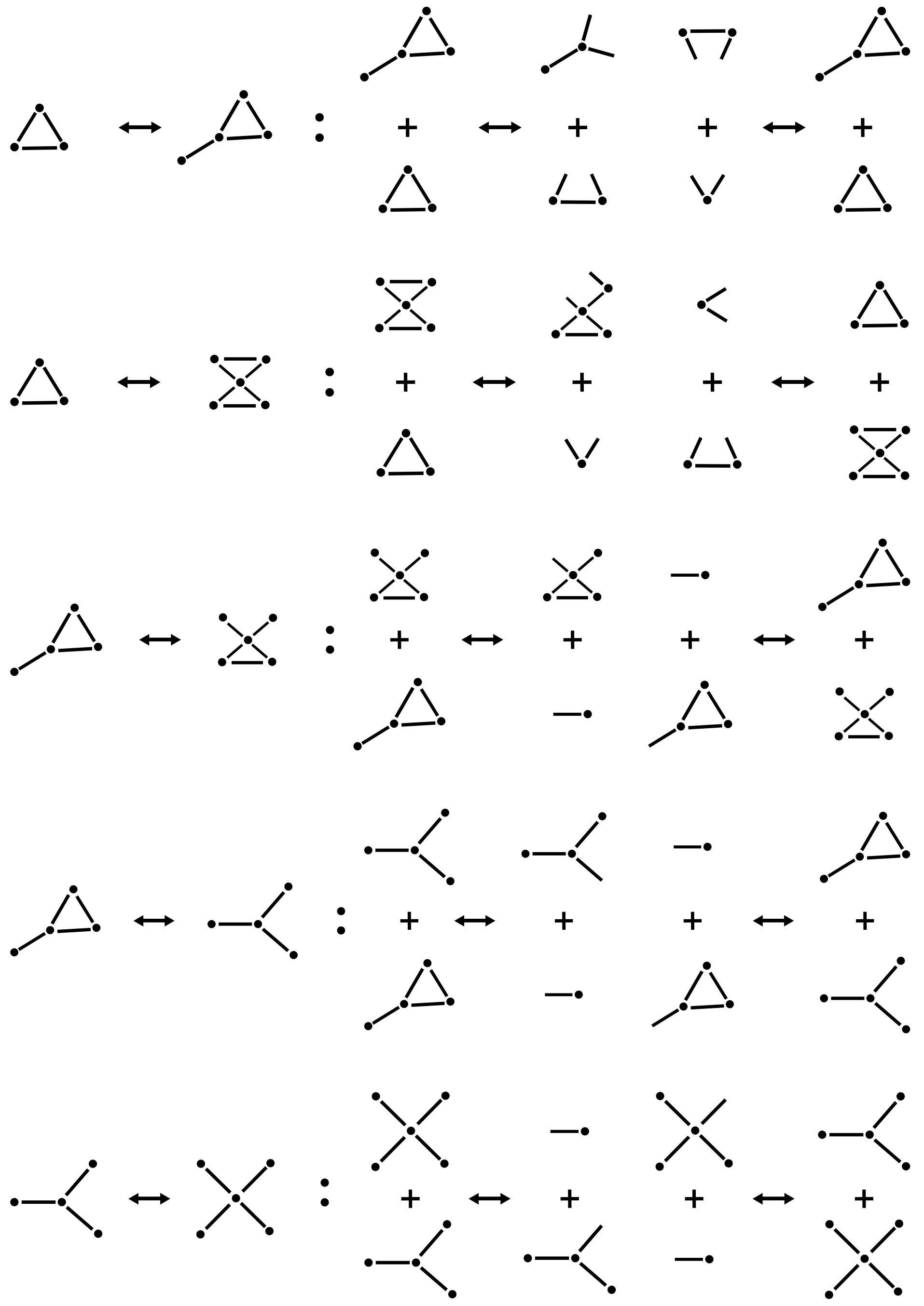

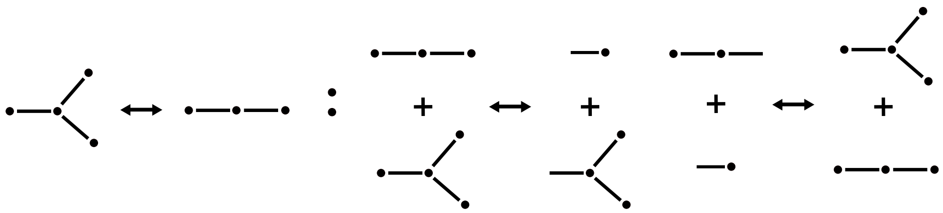

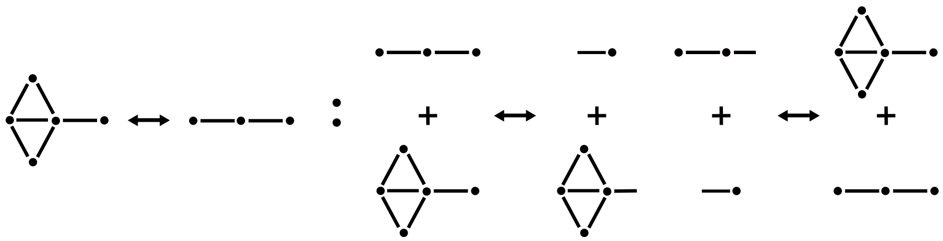

In summary, this yields:

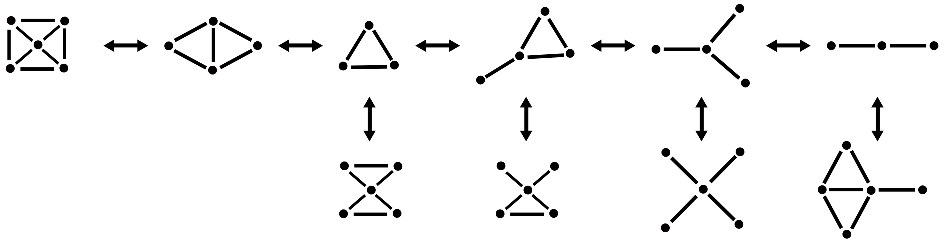

■
