# Supplementary material for: Crossover operators for molecular graphs with an application to virtual drug screening
Source: J Cheminform. 2025 Jun 17;17:97. doi: 10.1186/s13321-025-00958-w (PMC12175394; doi:10.1186/s13321-025-00958-w)

Additional file 2.

Subsets of the USPTO-10k “clean” set of patented molecules used in the benchmarking section.

Sample size: 1

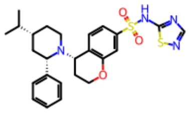

Sample size: 5

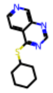
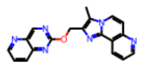
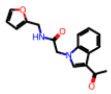
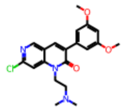
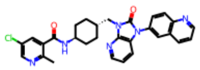

Sample size: 10

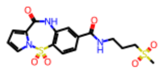
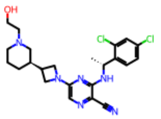
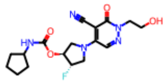
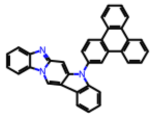
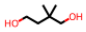

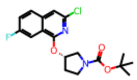
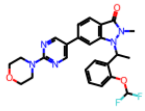
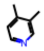
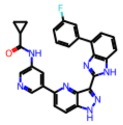
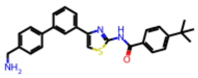

Supplement: Supplementary file 2 — Molecules used in the benchmarking section [file 13321_2025_958_MOESM2_ESM.pdf]
