# Supplementary material for: Crossover operators for molecular graphs with an application to virtual drug screening
Source: J Cheminform. 2025 Jun 17;17:97. doi: 10.1186/s13321-025-00958-w (PMC12175394; doi:10.1186/s13321-025-00958-w)
Supplement: Supplementary file 3 — Details on embedding violations observed for crossover products [file 13321_2025_958_MOESM3_ESM.pdf]

Additional file 3.

Details on embedding violations observed for crossover products.

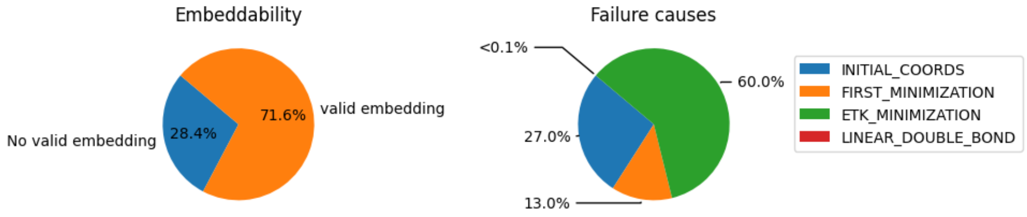

Left: A 3D embedding can be generated for the majority of offsprings generated from the structural formulas of molecules using natural cut-and-join crossover.

Right: For the 59,490 molecules that failed the embedding step, the failure causes were recorded. Most prominently the ETK_MINIMIZATION violation appears, which is raised if at least one atom that should be planar is not after minimization with the extended torsion or knowledge terms. The INITIAL_COORDS flag amounts to 27.0% of all failure cases, is raised if the random distance matrix is unable to generate initial coordinates. If the ETGK algorithm can not identify a conformation with sufficiently low energy the FIRST_MINIMIZATION violation is raised (Contributing in 13.0% of failures). If a double bonds substituent is in a linear geometry, LINEAR_DOUBLE_BOND is raised, which only amounts to a small fraction of less than 0.1% of recorded cases.

Explanations of the embedding failure flags were taken from the RDKIT blog `https://greglandrum.github.io/rdkit-blog/posts/2023-05-17-understanding-confgen-errors.html`
