# Supplementary material for: Crossover operators for molecular graphs with an application to virtual drug screening
Source: J Cheminform. 2025 Jun 17;17:97. doi: 10.1186/s13321-025-00958-w (PMC12175394; doi:10.1186/s13321-025-00958-w)
Supplement: Supplementary file 4 — Summary of MOSES statistics [file 13321_2025_958_MOESM4_ESM.pdf]

Additional file 4.

Summary of MOSES statistics

Metric	Recombinants			filtered			embedable		
	**1x**	**5x**	**10x**	**1x**	**5x**	**10x**	**1x**	**5x**	**10x**
**Samples**	4,697	30,000	30,000	2,241	30,000	30,000	2,148	28,605	30,000
**Validity**	100.0%	100.0%	100.0	100.0%	100.0%	100.0	100.0%	100.0%	100.0%
**Unique@1000**	1.0	1.0	1.0	1.0	1.0	1.0	1.0	1.0	1.0
**FCD**	39.32	21.48	22.2	40.6	21.95	22.68	40.61	22.41	20.36
**SNN**	0.44	0.45	0.43	0.46	0.5	0.47	0.46	0.51	0.5
**Frag**	0.19	0.44	0.21	0.2	0.45	0.23	0.2	0.45	0.29
**Scaf**	0.01	0.02	0.02	0.01	0.04	0.03	0.01	0.04	0.05
**IntDiv**	0.56	0.78	0.82	0.53	0.76	0.8	0.53	0.75	0.8
**IntDiv2**	0.54	0.75	0.79	0.51	0.73	0.77	0.51	0.72	0.77
**Filters**	0.17	0.32	0.25	0.17	0.34	0.23	0.17	0.33	0.27
**ΔlogP**	1.82	1.31	4.0	2.09	1.57	4.49	2.1	1.68	4.76
**ΔSA**	1.97	0.91	1.38	1.76	0.59	1.26	1.74	0.54	1.12
**ΔQED**	0.1	0.13	0.24	0.14	0.18	0.26	0.14	0.19	0.26
**ΔMW**	179.82	83.64	149.6	194.86	102.16	160.93	196.39	114.48	207.32

Molecular Sets (MOSES) benchmarking performed on the different recombinant sets generated from the USPTO-10k “clean” set. Each column represents the filtering step and every subcolumn referes to the sample size used in the crossover step. As recommended in [51], whenever possible a subset of 30000 samples were taken for the benchmarking.
